# Inhibiting calpain 1 and 2 in cyclin G associated kinase–knockout mice mitigates podocyte injury

**DOI:** 10.1172/jci.insight.142740

**Published:** 2020-11-19

**Authors:** Xuefei Tian, Kazunori Inoue, Yan Zhang, Ying Wang, C. John Sperati, Christopher E. Pedigo, Tingting Zhao, Meihua Yan, Marwin Groener, Dennis G. Moledina, Karen Ebenezer, Wei Li, Zhenhai Zhang, Dan A. Liebermann, Lois Greene, Peter Greer, Chirag R. Parikh, Shuta Ishibe

**Affiliations:** 1 Department of Internal Medicine, Yale University School of Medicine, New Haven, Connecticut, USA.; 2 State Key Laboratory of Organ Failure Research, Southern Medical University, Nanfang Hospital, Guangzhou, China.; 3 Center for Bioinformatics, School of Basic Medical Sciences, Southern Medical University, Guangzhou, China.; 4 Division of Nephrology, Johns Hopkins University School of Medicine, Baltimore, Maryland, USA.; 5 Fels Institute of Cancer Research and Molecular Biology and Department of Medical Genetics and Molecular Biochemistry, Lewis Katz School of Medicine, Temple University, Philadelphia, Pennsylvania USA.; 6 Laboratory of Cell Biology, National Heart, Lung, and Blood Institute, NIH, Bethesda, Maryland, USA.; 7 Queen’s Cancer Research Institute, Kingston, Ontario, Canada.

**Keywords:** Cell Biology, Nephrology, Chronic kidney disease, Mouse models

## Abstract

Evidence for reduced expression of cyclin G associated kinase (GAK) in glomeruli of patients with chronic kidney disease was observed in the Nephroseq human database, and GAK was found to be associated with the decline in kidney function. To examine the role of GAK, a protein that functions to uncoat clathrin during endocytosis, we generated podocyte-specific *Gak*-knockout mice (*Gak*-KO), which developed progressive proteinuria and kidney failure with global glomerulosclerosis. We isolated glomeruli from the mice carrying the mutation to perform messenger RNA profiling and unearthed evidence for dysregulated podocyte calpain protease activity as an important contributor to progressive podocyte damage. Treatment with calpain inhibitor III specifically inhibited calpain-1/-2 activities, mitigated the degree of proteinuria and glomerulosclerosis, and led to a striking increase in survival in the *Gak*-KO mice. Podocyte-specific deletion of *Capns1*, essential for calpain-1 and calpain-2 activities, also improved proteinuria and glomerulosclerosis in *Gak*-KO mice. Increased podocyte calpain activity–mediated proteolysis of IκBα resulted in increased NF-κB p65–induced expression of growth arrest and DNA-damage-inducible 45 beta in the *Gak*-KO mice. Our results suggest that loss of podocyte-associated *Gak* induces glomerular injury secondary to calcium dysregulation and aberrant calpain activation, which when inhibited, can provide a protective role.

## Abstract 2

Reduced cyclin G associated kinase (GAK) expression associates with diabetic and chronic kidney diseases, and GAK ablation in mice causes kidney failure due to calpain activation.

## Graphical abstract

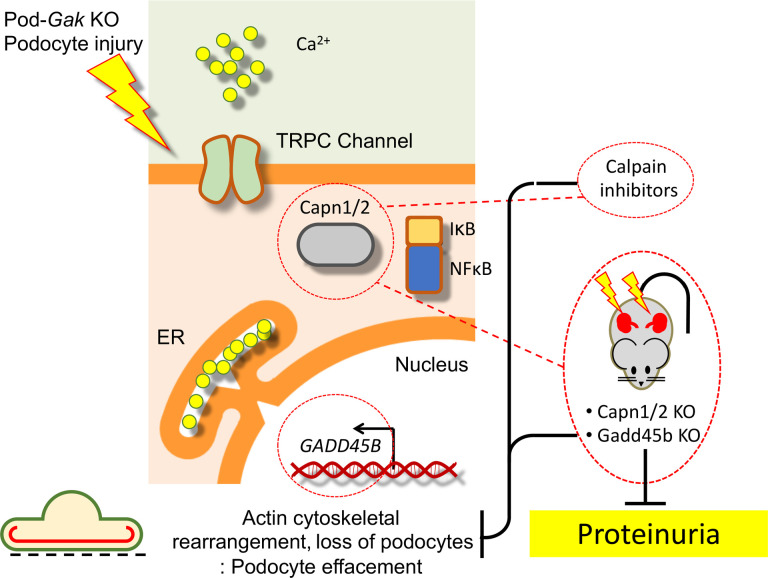

## Introduction

Dysfunction of the glomerular filtration barrier often manifests as nephrotic syndrome, a kidney disorder characterized by heavy proteinuria. Although glomerular diseases account for approximately 80% of chronic kidney disease (CKD) cases, the therapeutic interventions aiming to prevent the development or slow the progression are very limited. A better understanding of the molecular mechanisms underlying glomerular disease is necessary to develop more effective therapies. We have previously demonstrated that proteins critical for clathrin-mediated endocytosis — dynamin 1 and 2, synaptojanin1, and endophilin — are also critical to maintaining the glomerular filtration barrier (1). Recently, endocytic pathways have been implicated in human proteinuric diseases through identification of genetic mutations in *GAPVD1*, *ANKFY1*, and *TBC1D8B* (2, 3). To further investigate clathrin-mediated endocytosis in human disease, in this study we mined the Nephroseq v5 transcriptomic database. In 3 cohorts of patients with CKD, we observed a striking reduction in glomerular expression of cyclin G associated kinase (*GAK*), a ubiquitously expressed kinase that phosphorylates AP2 and has been associated with the uncoating of the clathrin coat (4, 5). In order to understand the pathobiology of *Gak* in glomerular disease, we next generated mice that exhibited podocyte-selective loss of *Gak*, using a podocin promoter-driven Cre recombinase transgenic and floxed *Gak* mouse lines. Our results showed that podocyte-specific *Gak*-deficient mice (*Gak*-KO mice) exhibited severe albuminuria, progressive glomerulosclerosis, kidney failure, foot process effacement, and decreased survival. To identify mechanisms of podocyte dysfunction in our model, we performed an unbiased transcriptomic screen using glomeruli from the *Gak*-KO mice. Our analysis of gene expression microarrays followed by calpain activity assays determined that calpain-1/-2 activation may play a critical role in mediating podocyte injury.

Calpains are a family of nonlysosomal cysteine proteases that are mainly activated by an increase in intracellular Ca^2+^ concentration (6–8). Within the calpain superfamily, 15 mammalian calpain gene products have been identified (9, 10). Calpain-1 (μ-form) and calpain-2 (m-form) are ubiquitously expressed calpain isoforms that exist as heterodimers consisting of an 80 kDa isoform-specific catalytic domain that are encoded by genes *Capn1* and *Capn2*, respectively, and a common 28 kDa regulatory domain (calpain small unit 1, CAPNS1 or CAPN4) encoded by *Capns1*, a gene that is indispensable for calpain-1/-2 stability and activity (11). Calpains have been shown to regulate cell behavior, actin cytoskeletal dynamics, cell motility, cell adhesion, endoplasmic reticulum stress, clathrin-dependent endocytosis, apoptosis, and inflammation upon interaction with their different substrates (12–14). We along with other groups have previously demonstrated that calpain activity is markedly increased in glomerular and urine samples from murine and human subjects with proteinuric kidney disease, while pursuant proteolysis of talin1, a critical protein linking the podocyte’s actin cytoskeletal architecture to the glomerular basement membrane, has been observed in injured glomeruli (13, 15, 16). To determine whether mitigating calpain activation in *Gak*-KO mice would inhibit disease progression, we administered a calpain inhibitor that inhibits calpain-1/-2 activities and observed reduced albuminuria, kidney failure, and death in the *Gak*-KO mice. Moreover, podocyte-specific deletion of *Capns1* in our *Gak*-KO mice was also associated with reduced kidney disease, further validating our calpain inhibitor studies. We also observed a calpain-mediated dysregulation of the NF-κB signaling pathway, resulting in a prominent increase in podocyte expression of growth arrest and DNA-damage-inducible 45 beta (*Gadd45b*), a transcription factor regulating cell survival, DNA damage repair, and cell cycle arrest (17) in the microarray from the *Gak*-KO glomeruli. Deletion of *Gadd45b* in the *Gak*-KO mice was also associated with reduced proteinuria and kidney failure, and increased survival correlated with reduced podocyte injury and stabilization of podocyte adhesion. These findings suggest that reducing aberrantly increased calpain-1/-2 activities in podocytes may provide a potentially novel therapeutic strategy for the treatment of proteinuric kidney diseases.

## Results

### Reduced glomerular GAK expression is observed in patients with impaired kidney function due to CKD and diabetic kidney disease.

**Figure 1:**
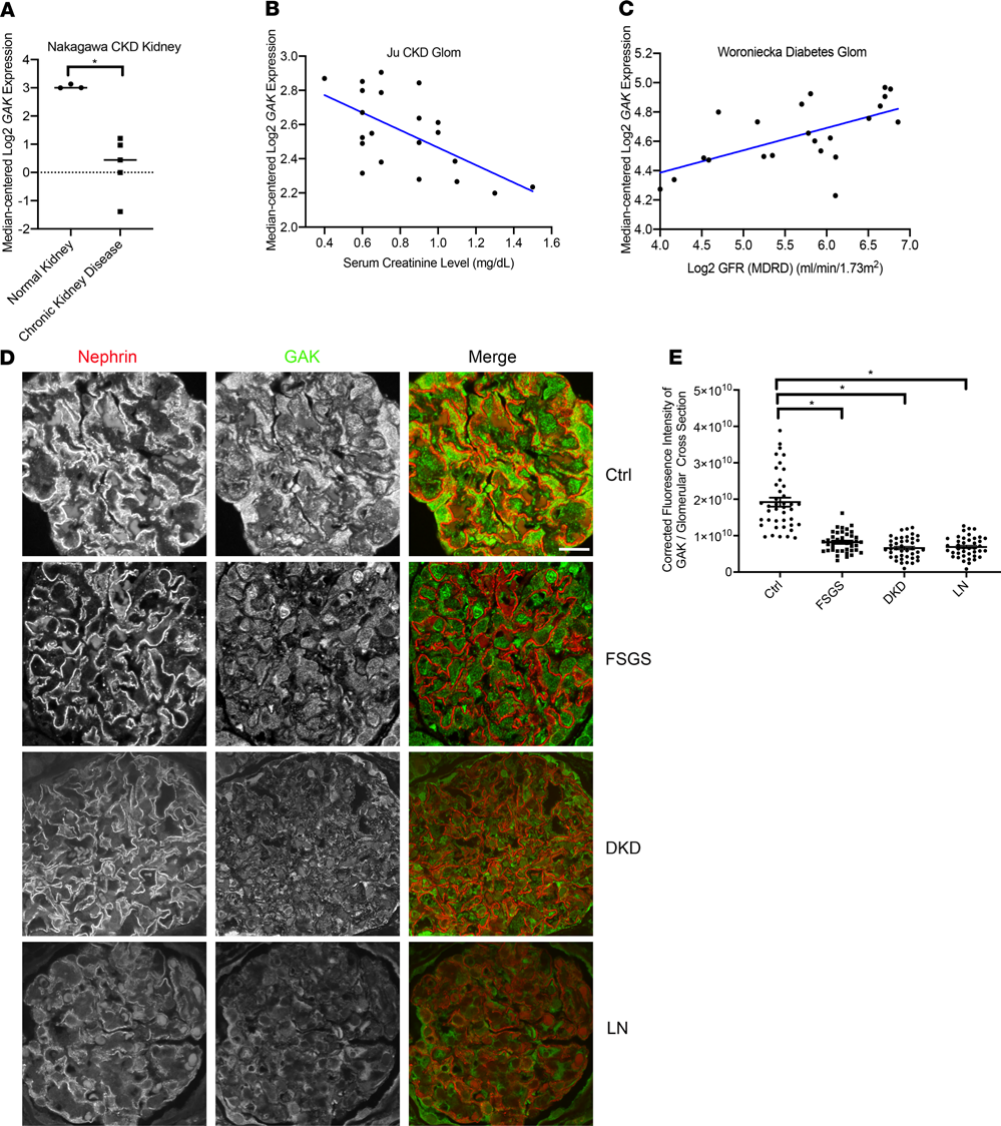
GAK expression is reduced in CKD glomeruli from Nephroseq data sets and human biopsies. (**A**) Reduced *GAK* expression in glomeruli from indicated cohort in the Nephroseq data set (fold change = –6.947, *P* = 0.004). (**B**) The association of glomerular *GAK* expression with serum creatinine level from a cohort of CKD patients in the Nephroseq data set (*r* = –0.607, *P* = 0.004). (**C**) The association of glomerular *GAK* expression with estimated GFR slope from a cohort of DKD patients in the Nephroseq data set (*r* = 0.605, *P* = 0.003). MDRD, Modification of Diet in Renal Disease. (**D**) Representative image of immunofluorescence of nephrin (red) and GAK (green) in control and FSGS human kidney biopsy specimens. Scale bar: 10 μm. (**E**) Quantification of **D** examining mean immunofluorescence intensity of GAK in the glomeruli *n* = 4 (controls, FSGS, DKD, LN), total 41 glomeruli from each group. *P < 0.01. Statistically analyzed via a 1-way ANOVA with Dunnett’s correction.

To ascertain the importance of proteins regulating clathrin-mediated endocytosis in human kidney disease, we interrogated the Nephroseq v5 transcriptomic database. We initially observed that clathrin-mediated pathways such as *DNM1*, *DNM2*, and *SH3GL2* (endophilin) were downregulated in microdissected glomeruli in this CKD cohort, which included glomerular diseases such as membranous nephropathy, lupus nephritis (LN), and diabetic kidney disease (Supplemental Figure 1, A–C; supplemental material available online with this article; https://doi.org/10.1172/jci.insight.142740DS1). Furthermore, we also observed a striking 6.947-fold reduction in *GAK* expression that correlated with a reduction in glomerular filtration rate (GFR) in this CKD cohort (Figure 1A), which was further validated in other CKD and diabetic kidney disease (DKD) cohorts in different data sets (Figure 1, B and C). These findings provided the impetus to further examine the importance of *GAK*. To determine if GAK transcript reduction was associated with reduced GAK protein expression in patients with CKD, we performed immunofluorescence on human focal segmental glomerulosclerosis (FSGS) biopsies costained for GAK and the podocyte-specific marker nephrin. We observed a striking reduction in GAK expression within the glomeruli (Figure 1D and quantified in Figure 1E).

### Loss of podocyte-associated Gak results in severe albuminuria, kidney failure, and death.

**Figure 2:**
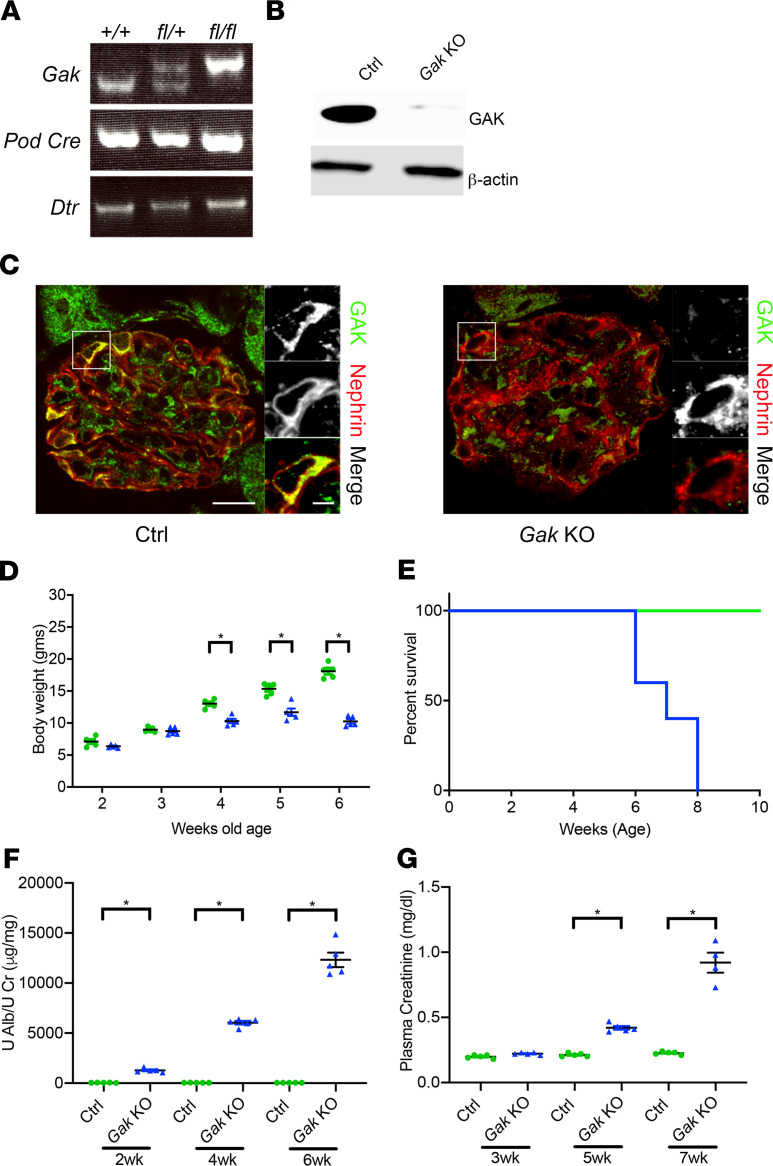
Generation of podocyte-specific *Gak*-KO mice results in severe proteinuria and kidney failure. (**A**) Identification of *Gak*, *Podocin-Cre*, and diphtheria toxin receptor (*Dtr*) by tail biopsy genotyping (age P5). (**B**) GAK expression in primary podocytes freshly isolated from control (Ctrl) mice and *Gak*-KO mice (age P7). (**C**) Double-immunofluorescence detection of GAK (green) and nephrin (red) on kidney sections of the control and *Gak*-KO mice (age P14). Scale bars: 10 μm. (**D**) *Gak*-KO mice fail to gain body weight by 4 weeks of age compared with control mice. *n* = 5 mice. *P < 0.05. (**E**) The survival curve of *Gak*-KO mice shows almost 80%–100% death by 8 weeks of age. *n* = 5 mice. (**F**) Quantification of urine albumin normalized to creatinine at 2, 4, and 6 weeks of age. *n* = 5 mice. *P < 0.05. (**G**) Elevated plasma creatinine in *Gak*-KO mice at 3, 5, and 7 weeks of age. *n* = 5 mice. *P < 0.05. Statistically analyzed via a 2-tailed *t* test.

Next, to examine if reduced *Gak* expression in podocytes contributes to albuminuria, we specifically deleted *Gak* in podocytes by crossing *Gak^fl/fl^* mice (4) with *Pod-Cre-Rosa-Dtr^fl/fl^* mice (18). *Gak*-KO mice were born at the expected Mendelian frequency, and mice carrying the mutation were verified by tail genotyping (Figure 2A), Western blot from isolated primary podocyte lysate (Figure 2B), and immunofluorescence of glomeruli from kidney sections (Figure 2C). The mutant mice appeared normal at birth, but failed to gain body weight after 4 weeks of age, compared with their *Gak^fl/fl^* littermate controls (Figure 2D). The *Gak*-KO mice exhibited albuminuria by 2 weeks of age (Figure 2F), a phenotype that progressed until 80%–100% of mice died by 8 weeks of age (Figure 2E) in the setting of kidney failure (Figure 2G).

### Loss of podocyte-associated Gak results in severe glomerulosclerosis and tubulointerstitial injury.

**Figure 3:**
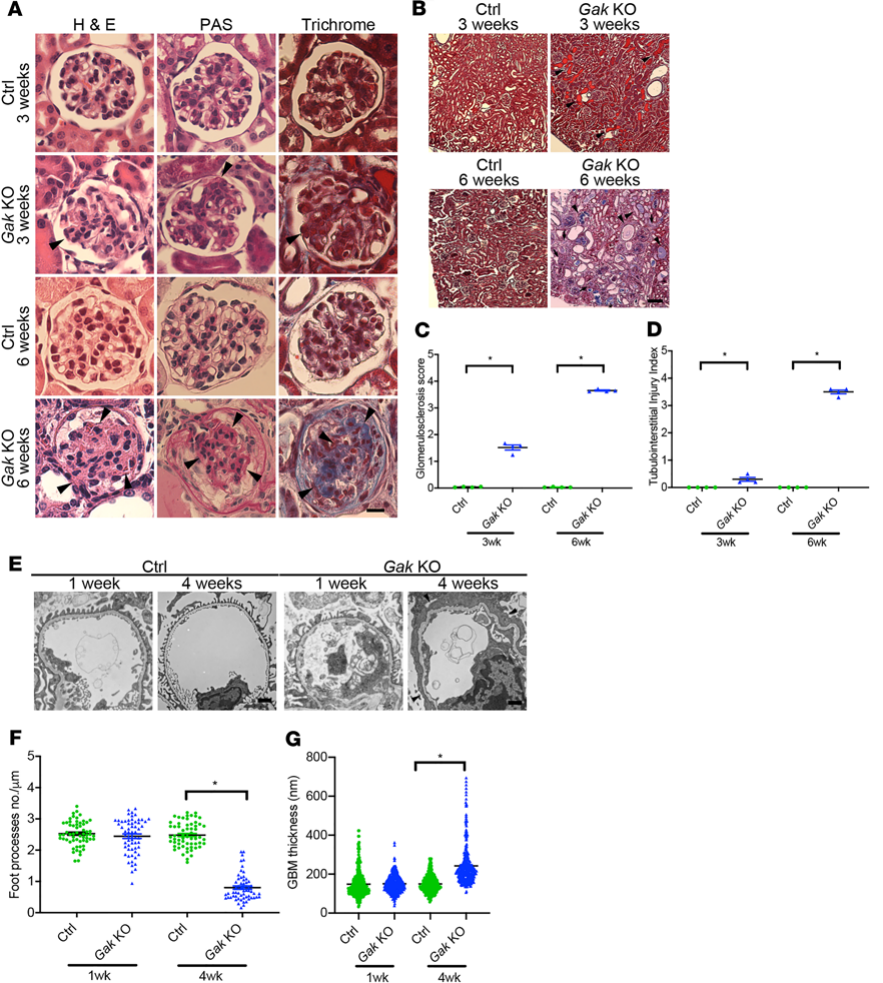
Podocyte-specific loss of *Gak* results in progressive glomerulosclerosis and tubulointerstitial fibrosis. (**A**) Representative light microscopy (H&E, periodic acid–Schiff [PAS], and Masson’s trichrome) of control mouse and *Gak*-KO mouse glomeruli at 3 weeks and 6 weeks of age. Arrowheads show mesangial matrix deposition and mesangial cell proliferation. Scale bar: 25 μm. (**B**) Representative trichrome staining in control and *Gak*-KO mouse kidneys at 3 and 6 weeks of age. Arrowheads depict dilated tubules, tubular atrophy, proteinaceous casts, and interstitial fibrosis. Scale bar: 100 μm. (**C**) Quantification of glomerulosclerosis in **A**. *n* = 4 mice. *P < 0.05 vs. control. (**D**) Quantification of tubulointerstitial injuries in **B**. *n* = 4 mice. *P < 0.05 vs. control. (**E**) Representative transmission electron micrograph (TEM) of foot processes in control and *Gak*-KO mouse at 1 and 4 weeks of age. Arrowheads depict podocyte foot process effacement. Scale bar: 1 μm. (**F**) Quantification of foot processes in **E**. *n* = 4 mice per group; 15 capillary tufts of glomeruli for each mouse were evaluated. *P < 0.05 vs. control. (**G**) Quantification of GBM thickness in **E**. *n* = 4 mice; GBM thickness from 326 random areas in each group was evaluated. *P < 0.05 vs. control. Statistically analyzed via a 2-tailed *t* test. In panels **F** and **G**, in each group, the large bar represents the mean, the small bar represents the SEM, and each dot represents 1 sample.

Histological analysis of kidneys from *Gak*-KO mice revealed progressive glomerulosclerosis (Figure 3A and quantified in Figure 3C) and tubulointerstitial injury, including tubular dilation, tubular atrophy, proteinaceous casts, and interstitial fibrosis (Figure 3B and quantified in Figure 3D). To further determine the ultrastructural changes in the glomerulus, transmission electron microscopy analysis was performed. Although there was no significant difference in the number of podocyte foot processes at 1 week of age between the *Gak*-KO and control mice, by 4 weeks of age, severe foot process effacement and thickened glomerular basement membrane (GBM) were observed in the *Gak*-KO mice (Figure 3E; quantified in Figure 3, F and G). These results suggest that *Gak* is indispensable for maintaining the integrity of the glomerular filtration barrier.

### mRNA profiling analysis suggests glomerular calpain-1/-2 activation in the Gak-KO mice.

**Figure 4:**
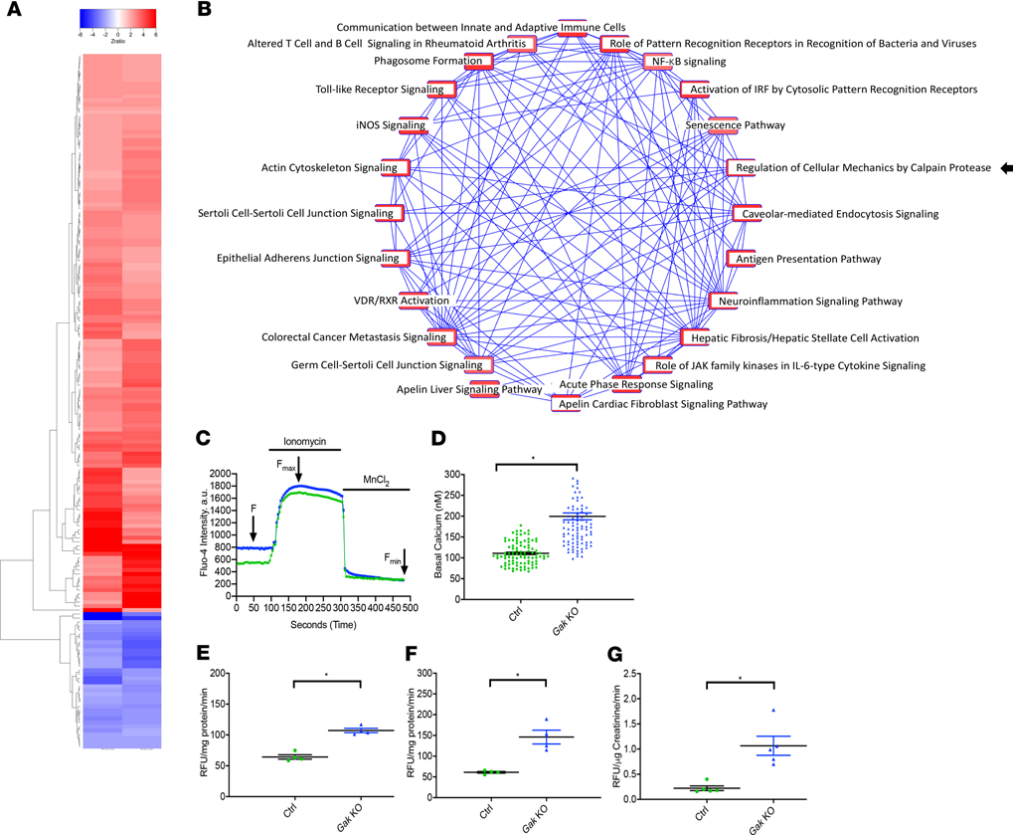
The increased activities of calpain-1 and calpain-2 are potential regulators in *Gak*-KO mice. (**A**) A two-dimensional hierarchical heatmap representing color-coded differentially expressed glomeruli genes analyzed by a *Z*-ratio of *Gak*-KO mice to control mice at 5 weeks of age. (**B**) Connectivity network map analyzing the candidate genes obtained from the glomerular DNA microarray using Ingenuity Pathway Analysis (IPA; QIAGEN) bioinformatics software. (**C**) Representative basal intracellular calcium imaging traces in control (green) and *Gak*-KO (blue) primary podocytes using Fluo-4 AM. The graphs show fluorescence signal changes in response to ionomycin. (**D**) Quantification of basal intracellular calcium levels in control (green) and *Gak*-KO mouse podocytes (blue). *n* = 4; 95 primary podocytes from each group of control and *Gak*-KO were evaluated. *P < 0.05 vs. control. (**E**) The calpain-1/-2 activities from the freshly isolated control mouse and *Gak*-KO mouse glomeruli at 5 weeks of age. *n* = 4. *P < 0.05 vs. control. (**F**) The calpain-1/-2 activities from isolated control mouse and *Gak*-KO mouse primary podocytes at P7. *n* = 4. *P < 0.05 vs. control. (**G**) The calpain-1/-2 activities from control and *Gak*-KO mouse urine samples at 5 weeks of age. *n* = 5. *P < 0.05 vs. control. Statistically analyzed via a 2-tailed *t* test.

Because *Gak*-KO mice developed severe albuminuria, glomerulosclerosis, and progressive kidney failure, we next sought to use an unbiased screen to identify differentially expressed genes (DEGs) that could explain this phenotype. Our analysis of gene expression microarrays yielded 139 statistically upregulated and 34 statistically downregulated genes from glomeruli harvested from *Gak*-KO mice as compared with littermate controls (Figure 4A) (Supplemental Table 1). Most DEGs could be categorized into 24 dysregulated pathways (Figure 4B). Among them, the top 5 dysregulated signaling pathways having the highest representation included hepatic fibrosis/hepatic stellate cell activation, neuroinflammation signaling pathway, acute phase response signaling, apelin cardiac fibroblast signaling pathways, and regulation of cellular mechanics by calpain protease. Analysis of the DEGs in these signaling pathways revealed the dysregulation of epithelial adherens junction signaling (19), actin cytoskeleton signaling (20), NF-κB signaling (21), and fibrosis pathway (22, 23) — all pathways regulated by calpain proteases. This suggested that calpain-1/calpain-2 may play a contributory role in this process. Therefore, we next isolated enriched primary podocytes using Rosa *Dtr^fl/fl^* mice, to obtain greater than 98% purity of primary podocytes after the addition of diphtheria toxin. Because calpain is a calcium-dependent, nonlysosomal cysteine protease, we assayed intracellular calcium concentration with Fluo-4 AM. Compared with control, *Gak*-KO podocytes displayed a striking increase in basal calcium concentration levels (representative tracings in Figure 4C and quantified in Figure 4D). Last, *Gak*-KO mice had increased calpain-1/-2 activities in the podocytes, glomeruli, and urine (Figure 4, E–G), which mirrors similar findings in human FSGS patients (13, 15). These results suggest that increased podocyte calpain-1/-2 activities may play a prominent role in the progression of glomerular dysfunction observed in the *Gak*-KO mice.

### Calpain inhibitor treatment reduces albuminuria and progression of glomerular injury.

**Figure 5:**
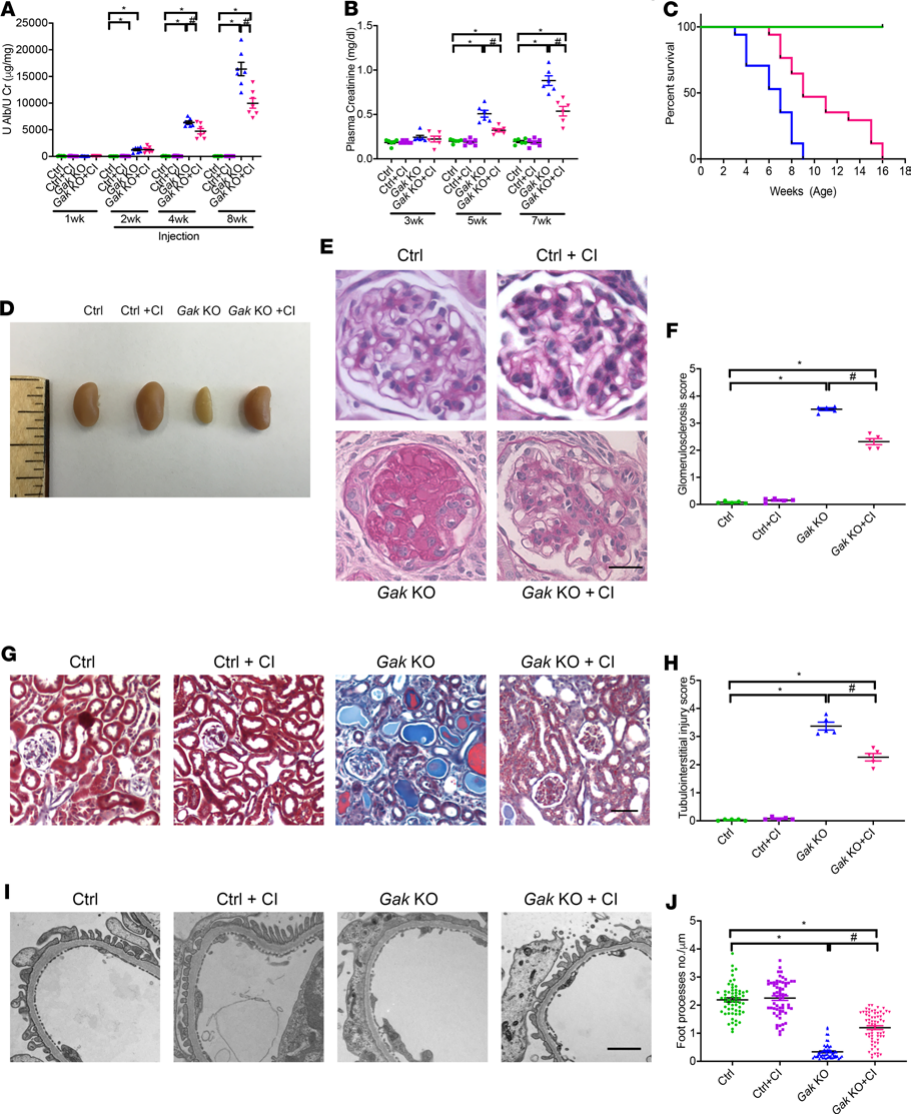
CI inhibits the calpain-1/-2 activities and reduces podocyte injury in *Gak*-KO mice. (**A**) Urine albumin/creatinine ratio in control (green), control+CI (purple), *Gak*-KO (blue), and *Gak*-KO+CI (red) mice at 1, 2, 4, and 8 weeks of age; treatment started with vehicle or CI from 2 weeks of age. *n* = 7. *P < 0.05 vs. control mice, *^#^P* < 0.05 vs. *Gak*-KO mice. (**B**) Plasma creatinine in control, control+CI, *Gak*-KO, and *Gak*-KO+CI mice at 3, 5, and 7 weeks of age. *n* = 6. *P < 0.05 vs. control mice; ^#^*P* < 0.05 vs. *Gak*-KO mice. (**C**) Survival curves of control, control+CI, *Gak*-KO, and *Gak*-KO+CI mice. *n* = 17. (**D**) Kidneys of *Gak*-KO mice were corrugated, paler, and smaller than those of control, control+CI, and *Gak* +CI mice at 7 weeks of age. (**E**) Representative light microscopy images of PAS staining in control, control+CI, *Gak*-KO, and *Gak*-KO+CI mice at 7 weeks of age. Scale bar: 25 μm. (**F**) Glomerulosclerosis quantification of **E**. *n* = 5. *P < 0.05 vs. control mice; ^#^*P* < 0.05 vs. *Gak*-KO mice. (**G**) Representative images of trichrome staining in control, control+CI, *Gak*-KO, and *Gak*-KO+CI mice at 7 weeks of age. Scale bar: 100 μm. (**H**) Tubulointerstitial injury quantification of **G**. *n* = 5 as shown on the graph. *P < 0.05 vs. control mice; ^#^*P* < 0.05 vs. *Gak*-KO mice. (**I**) Representative TEM images in control, control+CI, *Gak*-KO, and *Gak*-KO+CI mice at 7 weeks of age. Scale bar: 1 μm. (**J**) Quantification of foot process effacement in **I**. *n* = 4 mice per group; 15 capillary tufts of glomeruli for each mouse were evaluated. *P < 0.05 vs. control mice; ^#^*P* < 0.05 vs. *Gak*-KO mice (**A**, **B**, **F**, **H**, and **J**). Statistically analyzed via a 1-way ANOVA with Dunnett’s correction.

Next, we examined whether inhibition of the aberrant calpain activation would stabilize albuminuria and mitigate the progression of glomerular injury. *Gak*-KO mice were treated with calpain inhibitor III (CI) starting at 2 weeks of age, when the mice displayed evident albuminuria. CI is a potent, cell-permeable pharmacological compound specifically inhibiting calpain-1/-2. The treatment resulted in a striking reduction in albuminuria (Figure 5A). Furthermore, previously observed elevations of plasma creatinine were also mitigated in the treated *Gak*-KO mice (Figure 5B). The mutant mice treated with CI also displayed increased survival (Figure 5C) and maintenance of body weight (data not shown) when compared with the untreated *Gak*-KO mice. Moreover, compared with *Gak*-KO mice, following CI treatment, the kidneys were grossly normal without corrugation or fibrotic appearance (Figure 5D). Furthermore, histological analysis showed improvement in glomerulosclerosis (Figure 5E, quantified in Figure 5F), tubular dilation, and interstitial fibrosis (Figure 5G, quantified in Figure 5H) in the *Gak*-KO mice treated with CI. Transmission electron microscopy analyses demonstrated reduced foot process effacement in the *Gak*-KO mice treated with CI when compared with untreated mutant mice (Figure 5I and quantified in Figure 5J). No observed side effects in mice were observed following treatment with CI in controls.

To evaluate whether calpain-1/-2 activation was also observed in a murine podocyte injury model, we injected anti-murine GBM rabbit antiserum (nephrotoxic serum, NTS) (23) into 8-week-old control mice. There was a marked increase in calpain-1/-2 activities in the isolated glomeruli (Supplemental Figure 2A) and urine samples in NTS-treated mice (data not shown) after 7 and 14 days compared with vehicle-treated control mice. Administration of CI starting 2 days post-NTS markedly reduced glomerular calpain-1/-2 activities (Supplemental Figure 2B), albuminuria (data not shown), mesangial matrix accumulation, and tubulointerstitial injury (Supplemental Figure 2, C and D, and quantified in Supplemental Figure 2, E and F). These results indicate inhibition of elevated calpain-1/-2 activities may be beneficial following podocyte injury.

### Loss of Capns1 in podocytes improves glomerulosclerosis and tubulointerstitial injury in the Gak-KO mice.

**Figure 6:**
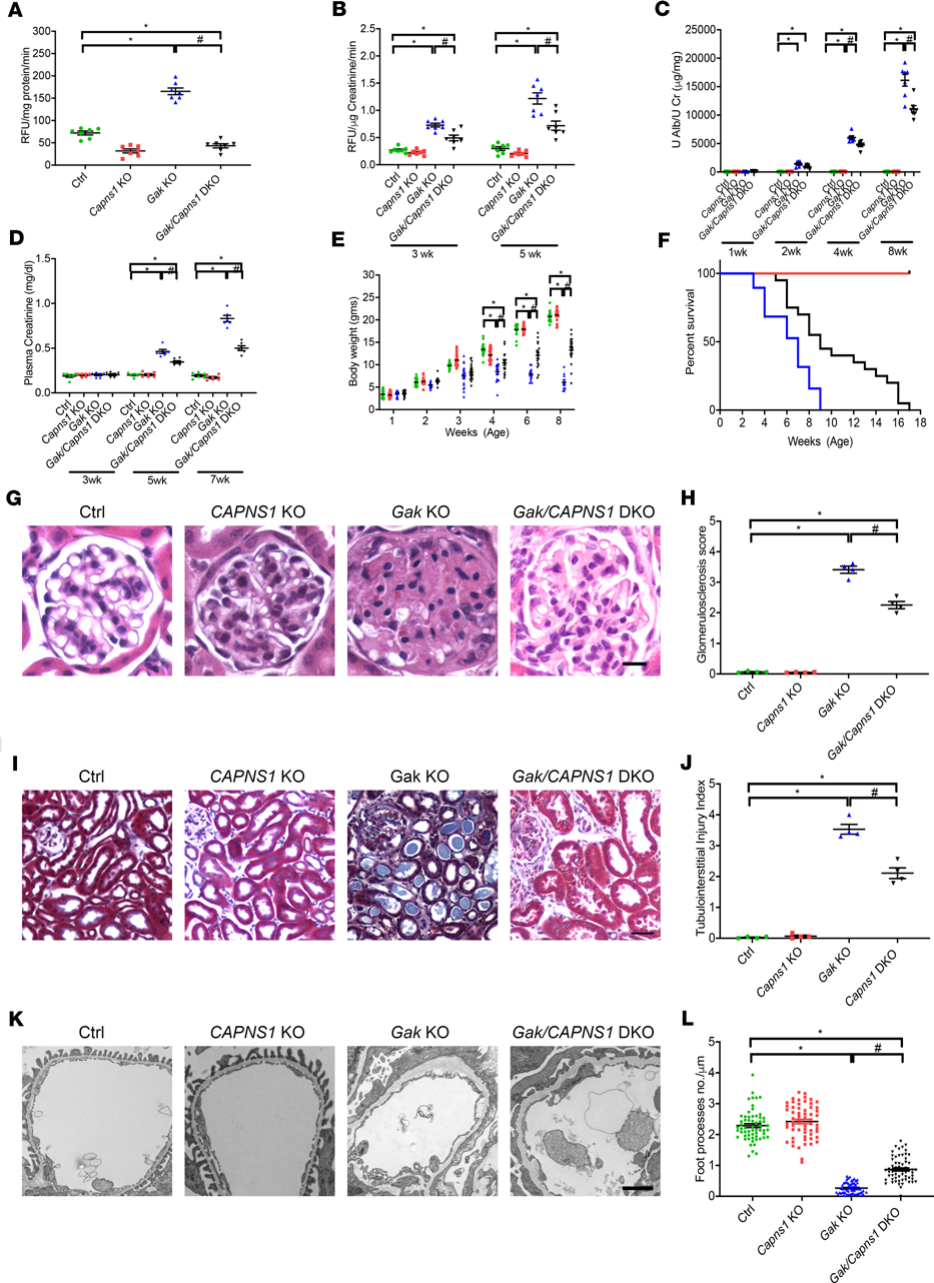
Podocyte deletion of *Capns1* improves glomerulosclerosis and kidney failure in *Gak*-KO mice. (**A**) Calpain-1/-2 activities in primary podocytes isolated from control (green), *Capns1*-KO (red), *Gak*-KO (blue), and *Gak/Capns1*-DKO (black) mice at P14. *n* = 7. *P < 0.05 vs. control mice; ^#^*P* < 0.05 vs. *Gak*-KO mice. (**B**) Calpain-1/-2 activities in urine samples from control (green), *Capns1*-KO (red), *Gak*-KO (blue), and *Gak/Capns1*-DKO (black) mice at 3 weeks of age and 5 weeks of age. *n* = 7. *P < 0.05 vs. control mice; ^#^*P* < 0.05 vs. *Gak*-KO mice. (**C**) Quantification of urine albumin/creatinine ratio at 1, 2, 4, and 8 weeks of age from control (green), *Capns1*-KO (red), *Gak*-KO (blue), and *Gak/Capns1*-DKO (black) mice. *n* = 7 as shown on the graph. *P < 0.05 vs. control mice; ^#^*P* < 0.05 vs. *Gak*-KO mice. (**D**) Plasma creatinine levels at 3, 5, and 7 weeks of age from control (green), *Capns1*-KO (red), *Gak*-KO (blue), and *Gak/Capns1*-DKO (black) mice. *n* = 6. *P < 0.05 vs. control mice; ^#^*P* < 0.05 vs. *Gak*-KO mice. (**E**) Body weight at 1, 2, 3, 4, 6, and 8 weeks of age from control (green), *Capns1*-KO (red), *Gak*-KO (blue), and *Gak/CapnS1*-DKO (black) mice. *n* = 21. *P < 0.05 vs. control mice; ^#^*P* < 0.05 vs. *Gak*-KO mice. (**F**) Survival curves from control (green), *Capns1*-KO (red), *Gak*-KO (blue), and *Gak/Capns1*-DKO (black) mice. *n* = 19. (**G**) Representative light microscopy images (H&E) from control, *Capns1*-KO, *Gak*-KO, and *Gak/CAPNS1*-DKO mice kidneys at 7 weeks of age. Scale bar: 25 μm. (**H**) Quantification of glomerulosclerosis in **G**. *n* = 4. *P < 0.05 vs. control mice; ^#^*P* < 0.05 vs. *Gak*-KO mice. (**I**) Representative trichrome staining from control, *Capns1*-KO, *Gak*-KO, and *Gak/Capns1*-DKO mouse kidneys at 7 weeks of age. Scale bar: 100 μm. (**J**) Quantification of tubulointerstitial injuries in **I**. *n* = 4. *P < 0.05 vs. control mice; *^#^P* < 0.05 vs. *Gak*-KO mice. (**K**) Representative TEM images from control, *Capns1*-KO, *Gak*-KO, and *Gak/Capns1*-DKO mouse kidneys at 5 weeks of age. Scale bar: 1 μm. (**L**) Quantification of foot processes in **K**. *n* = 4 mice per group; 15 capillary tufts of glomeruli for each mouse were evaluated. (**A**–**E**, **H**, **J**, and **L**) Statistically analyzed via a 1-way ANOVA with Dunnett’s correction.

To further clarify the mechanism and site of action of the CI in this model of inhibition of podocyte-associated calpain-1/-2 activities, we next crossed the *Capns1^fl/fl^* mice with the *Pod-Cre-Rosa-Dtr^fl^* mice to generate a podocyte-specific *Capns1*-deletion mouse (*Capns1*-KO), which was confirmed by Western blot and immunofluorescence (Supplemental Figure 3, A–C). *Capns1*-KO mice were devoid of any kidney phenotype for greater than 18 months of age as characterized by urine albumin, plasma creatinine, and kidney histology in comparison with littermate controls (data not shown). We next examined whether loss of *Capns1* could rescue podocyte injury in the *Gak*-KO mice, by generating podocyte-specific *Gak*/*Capns1*–double knockout (*Gak*/*Capns1*-DKO) mice. We initially confirmed that loss of podocyte-associated *Capns1* reduced calpain-1/-2 activities in the *Gak*-KO mice podocytes (Figure 6A) and in the urine (Figure 6B). Phenocopying what was found in the inhibitor studies, we observed improved urinary albumin/creatinine ratios (Figure 6C), kidney function (Figure 6D), body weight (Figure 6E), and survival (Figure 6F), when compared with the *Gak*-KO mice. Histological analysis in the *Gak/Capns1*-DKO mice revealed a reduction in glomerulosclerosis (Figure 6G and quantified in Figure 6H) and tubulointerstitial injury (Figure 6I and quantified in Figure 6J). Ultrastructural examination also demonstrated reduced foot process effacement in the *Gak/Capns1*-DKO mice (Figure 6K and quantified in Figure 6L). To further validate the protective role of the loss of podocyte-associated *Capns1*, we induced NTS nephritis (23) using control and *Capns1*-KO mice. Following NTS injection, reductions in albuminuria (Supplemental Figure 4A) and podocyte injury (Supplemental Figure 4B and quantified in Supplemental Figure 4C) were observed in mice lacking *Capns1* in their podocytes. These results demonstrate that following loss of *Gak*, mitigating calpain-1/-2 activation in a podocyte-specific manner may prove to be beneficial in reducing progression of glomerular disease.

### Loss of Capns1 results in increased GADD45B expression and IκBα cleavage.

**Figure 7:**
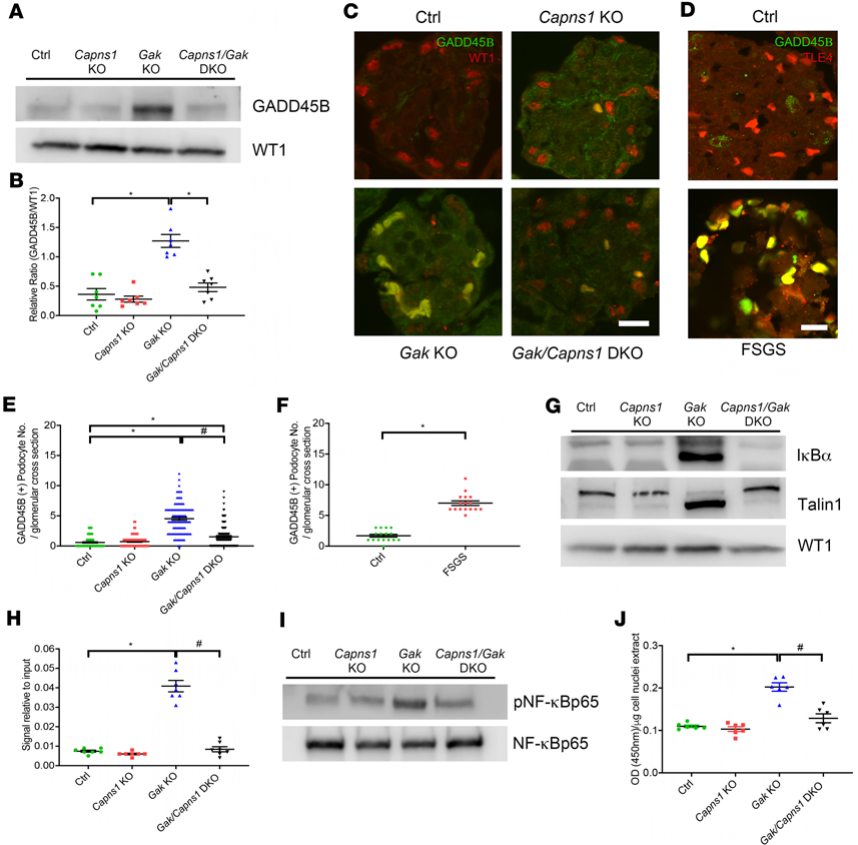
Loss of *Capns1* results in increased GADD45B expression due to IκBα cleavage and NF-κB p65 signaling in *Gak*-KO mice. (**A**) Representative Western blot images from control, *Capns1*-KO, *Gak*-KO, and *Gak/CapnsS1*-DKO mouse primary podocytes immunoblotted with GADD45B and Wilms tumor 1 (WT1). (**B**) Quantification of **A** by densitometry. *n* = 6. *P < 0.05**.** (**C**) Representative immunofluorescence image of *Gak*-KO mouse glomeruli demonstrates increased GADD45B expression (GADD45B in green and WT1 in red). Scale bar: 50 μm. (**D**) Representative immunofluorescence image of glomeruli from control and FSGS patient stained with GADD45B (green) and TLE4 (red). Scale bar: 10 μm. (**E**) Quantification of **C**. *n* = 5 mice; 20 glomeruli per mouse were evaluated. (**F**) Quantification of **D**. *n* = 3 patients; 16 glomeruli per group were evaluated. (**G**) *Gak*-KO mice podocytes show IκBα cleavage (upper panel) and talin1 cleavage (middle panel) as determined by Western blots; these cleavage events were reduced by the podocyte-specific loss of *Capns1*. (**H**) ChIP assay using phospho-NF-κB p65 and primer sets for *Gadd45b* promoter in the primary podocytes of control (green), *Capns1*-KO (red), *Gak*-KO (blue), and *Gak/Capns1*-DKO (black) mice. *n* = 7. (**I**) Representative Western blot images for phospho-NF-κB p65 (pNF-κB p65) (Ser536) and total NF-κB p65 (NF-κB p65) from primary podocytes in control, *Capns1*-KO, *Gak*-KO, and *Gak/Capns1*-DKO mice. (**J**) Quantification of the nuclear NF-κB p65 activities of primary podocytes isolated from control (green), *Capns1*-KO (red), *Gak*-KO (blue), and *Gak/Capns1*-DKO (black) mice at the age of P14. *n* = 6. *P < 0.05 vs. control mice; *^#^P* < 0.05 vs. *Gak*-KO mice. (**E**, **F**, **H**, and **J**). Statistically analyzed via a 1-way ANOVA with Dunnett’s correction.

To further investigate the downstream mechanisms that are regulated by calpain-1/-2 activation, we revisited the microarray data from our *Gak*-KO mice glomeruli samples. We confirmed by reverse transcriptase PCR that 25 genes with *Z*-ratios greater than 2 were potentially associated with podocyte function and cellular maintenance based on the publications and network information available. Of these 25 genes, *Gadd45b* was highly upregulated in *Gak*-KO mouse glomeruli compared with littermate control glomeruli (Supplemental Figure 5). The increased expression of GADD45B in *Gak*-KO primary podocytes was confirmed initially by Western blot and was mitigated in the *Gak*/*Capns1*-DKO podocytes (Figure 7A and quantified in Figure 7B). Immunofluorescence also demonstrated increased podocyte GADD45B expression both in *Gak*-KO mouse glomeruli (Figure 7C and quantified in Figure 7E) and in biopsies from human FSGS patients (Figure 7D and quantified in Figure 7F) when compared with control kidney samples. To next examine how GADD45B expression was increased following podocyte injury, we revisited our IPA bioinformatic analysis on mouse glomeruli samples, which indicated a dysregulated NF-κB signaling pathway in the *Gak*-KO mice. IκBα is an important calpain substrate, and proteolysis of IκBα results in NF-κB activation (24–26), which in turn has been shown to induce increased *Gadd45b* expression (27). Western blot results demonstrated a marked cleavage of IκBα in *Gak*-KO podocytes compared with littermate control podocytes; this effect was attenuated in the *Gak/Capns1*-DKO podocytes (Figure 7G). NF-κB p65 binds to the *Gadd45b* promoter to induce its expression (25). A chromatin immunoprecipitation (ChIP) assay demonstrated an increased binding of phospho-NF-κB p65 to the *Gadd45b* promoter in *Gak*-KO podocytes when compared with the control podocytes (Figure 7H). In *Gak*-KO primary podocytes, we also noted increased NF-κB p65 activity (Figure 7I) as well as increased phosphorylation of NF-κB p65 (Ser536) (Figure 7J), which was attenuated in the *Gak/Capns1*-DKO podocytes. These results suggest that loss of *Gak* in podocytes potentiates calpain-1/-2 activities, which induces the NF-κB signaling pathway through calpain-mediated IκBα cleavage, resulting in a subsequent increase in GADD45B expression.

### Loss of Gadd45b results in reduced albuminuria, kidney failure, and glomerulosclerosis.

**Figure 8:**
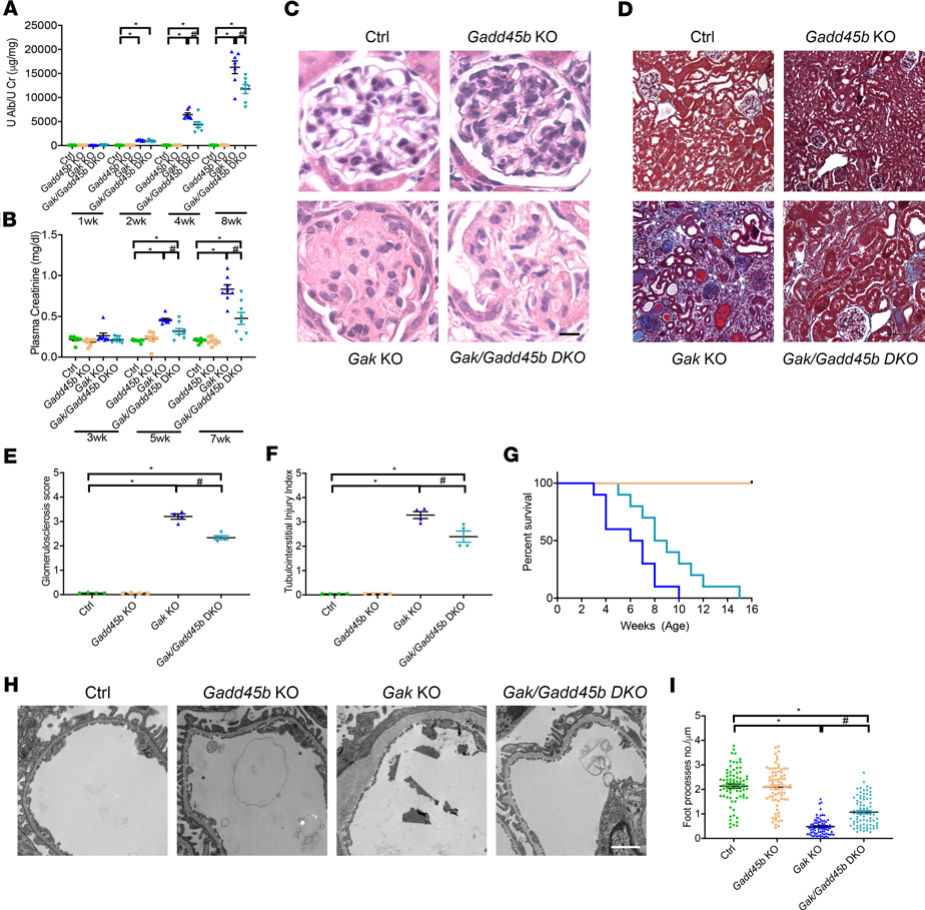
Loss of *Gadd45b* in *Gak* mice improves glomerulosclerosis and tubulointerstitial injury. (**A**) Urine albumin/creatinine ratio from control (green), *Gadd45b*-KO (yellow), *Gak*-KO (blue), and *Gak/Gadd45b*-DKO (light blue) mice at 1, 2, 4, and 8 weeks of age. *n* = 7. *P < 0.05 vs. control mice; ^#^*P* < 0.05 vs. *Gak*-KO mice. (**B**) Plasma creatinine from control, *Gadd45b*-KO, *Gak*-KO, and *Gak/Gadd45b*-DKO mice at 3, 5, and 7 weeks of age. *n* = 8. *P < 0.05 vs. control mice; ^#^*P* < 0.05 vs. *Gak*-KO mice. (**C**) Representative light microscopy images of PAS staining from control, *Gadd45b*-KO, *Gak*-KO, and *Gak/Gadd45b*-DKO mice at 7 weeks of age. Scale bar: 25 μm. (**D**) Representative trichrome staining in control, *Gadd45b*-KO, *Gak*-KO, and *Gak/Gadd45b-*DKO mice at 7 weeks of age. Scale bar: 100 μm. (**E**) Quantification of glomerulosclerosis in **C**. *n* = 4. *P* < 0.05 vs. control mice; ^#^*P* < 0.05 vs. *Gak*-KO mice. (**F**) Tubulointerstitial injury quantification in **D**. *n* = 4. *P < 0.05 vs. control mice; ^#^*P* < 0.05 vs. *Gak*-KO mice. (**G**) Survival curves from control (green), *Gadd45b*-KO (yellow), *Gak*-KO (blue), and *Gak/Gadd45b*-DKO (black) mice. *n* = 8. There were no deaths in control and *Gadd45b*-KO groups. (**H**) Representative TEM images in control, *Gadd45b*-KO, *Gak*-KO, and *Gak/Gadd45b*-DKO mice at 5 weeks of age. Scale bar: 1 μm. (**I**) Foot processes quantification in **H**. *n* = 4 mice per group; 19 capillary tufts from glomeruli of each mouse were evaluated. In each group, the large bar represents the mean, the small bar represents the SEM, and each dot represents 1 sample. *P < 0.05 vs. control mice; ^#^*P* < 0.05 vs. *Gak*-KO mice. (**A**) Statistically analyzed via a 2-tailed *t* test. (**B**, **C**, **E**, **G**, and **I**) Statistically analyzed via a 1-way ANOVA with Dunnett’s correction.

To determine the role of *Gadd45*b in *Gak*-KO mice, we next generated *Gak*/*Gadd45b*-DKO mice. Compared with *Gak*-KO mice, we observed a striking reduction in albuminuria (Figure 8A), kidney failure (Figure 8B), glomerulosclerosis (Figure 8C and quantified in Figure 8E), tubulointerstitial injury (Figure 8D and quantified in Figure 8F), and foot process effacement in *Gak*/*Gadd45b*-DKO mice (Figure 8H and quantified in Figure 8I). The *Gak*/*Gadd45b*-DKO mice also displayed prolonged survival (Figure 8G) and maintained their normalized body weight (data not shown). These results recapitulated the observed protective effects of deleting *Capns1* in *Gak*-KO podocytes, suggesting that like calpain, *Gadd45b* also has a kidney disease–promoting role in *Gak*-KO podocyte mice.

### Loss of Capns1 in podocytes improves the disorganized cytoskeletal structure and adhesion in the Gak-KO mice.

**Figure 9:**
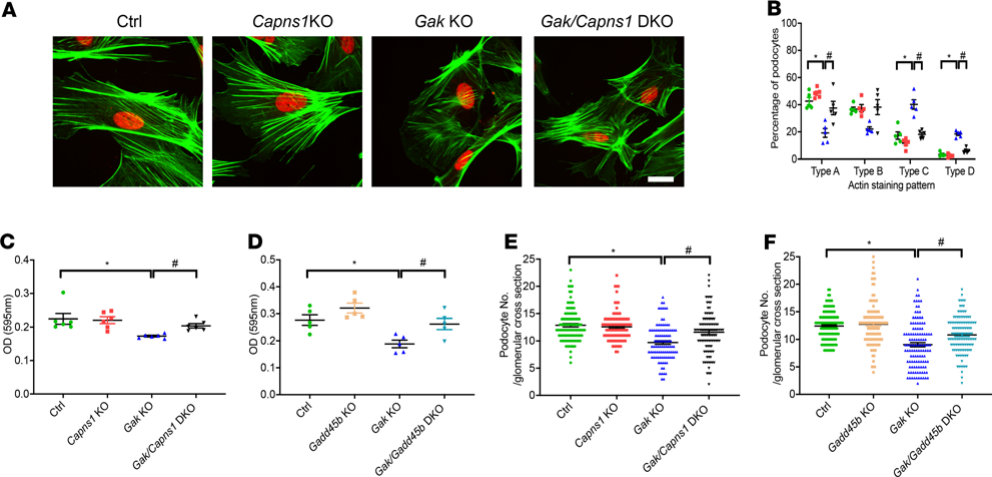
Lack of podocyte *Capns1* or *Gadd45b-*KO in *Gak*-KO mice improves podocyte numbers and adhesion. (**A**) Representative immunostaining for phalloidin (green) and WT1 (red) from control, *Capns1*-KO, *Gak*-KO, and *Gak/Capns1*-DKO mice. Scale bar: 10 μm. (**B**) Quantification of phalloidin staining patterns from control (green), *Capns1*-KO (red), *Gak*-KO (blue), and *Gak/Capns1*-DKO (black) mice primary podocytes in **A**. *n* = 5. (**C**) Adhesion of primary podocytes isolated from control (green), *Capns1*-KO (red), *Gak*-KO (blue), and *Gak/Capns1*-DKO (black) mice at P14. *n* = 6. *P < 0.05 vs. control mice; ^#^*P* < 0.05 vs. *Gak*-KO mice. (**D**) Adhesion of primary podocytes isolated from control (green), *Gadd45b*-KO (yellow), *Gak*-KO (blue), and *Gak/Gadd45b*-DKO (light blue). *n* = 5. *P < 0.05 vs. control mice; ^#^*P* < 0.05 vs. *Gak*-KO mice. (**E**) Quantification of WT1 staining per glomerulus from control, *Capns1*-KO, *Gak*-KO, and *Gak/Capns1*-DKO mice. *n* = 5 mouse kidney sections; 100 total glomeruli from each group were evaluated. *P < 0.05 vs. control mice; ^#^*P* < 0.05 vs. *Gak*-KO mice. (**F**) Quantification of WT1 staining per glomerulus in control, *Gadd45b*-KO, *Gak*-KO, and *Gak/Gadd45b*-DKO mice. *n* = 5 mice kidney sections; 120 total glomeruli each group were evaluated. *P < 0.05 vs. control mice; ^#^*P* < 0.05 vs. *Gak*-KO mice. (**B**, **D**, **E**, and **F**) Statistically analyzed via a 1-way ANOVA with Dunnett’s correction. In panels **E** and **F**, in each group, the large bar represents the mean, the small bar represents the SEM, and each dot represents 1 sample.

Aberrant calpain activation has been shown to dysregulate important actin-related molecules, such as talin, vinculin, paxillin, and focal adhesion kinase (28, 29). It has been postulated that podocyte injury results in foot process effacement through actin rearrangement (30). The immunoblotting results showed a marked cleavage of talin1 in *Gak*-KO podocytes that was mitigated in the *Gak/Capns1*-DKO podocytes (Figure 7F, middle panel). Immunostaining with phalloidin (F-actin) in *Gak*-KO podocytes showed significantly disorganized actin stress fibers. This aberration was significantly improved in *Gak/Capns1*-DKO podocytes (Figure 9A and quantified in Figure 9B).

Because podocyte adherence to the GBM is critical to maintain a functioning glomerular filtration barrier (31), we next questioned whether the loss of *Gak* affects the adherence properties using cultured primary podocytes. *Gak*-KO podocytes demonstrated decreased adhesion compared with podocytes from littermate controls. This adhesion phenotype was rescued by concurrent *Capns1* deletion in the *Gak*-KO podocytes (Figure 9C). Similarly, the loss of *Gadd45b* in the *Gak/Gadd45b*-DKO rescued the Gak-KO phenotype adhesion defect (Figure 9D). The podocyte density in *Gak*-KO mice kidney sections was next determined by staining for WT1, a podocyte-specific cell marker, and imaged via immunofluorescence to quantify total podocyte number in the control, *Gak*-KO, *Gak/Capns1*-DKO, and *Gak*/*Gadd45b*-DKO mice. The results demonstrated a marked decrease in WT1-positive podocytes in the *Gak*-KO mouse glomeruli when compared with the control conditions. Despite this, total podocyte numbers were significantly higher in both the *Gak/Capns1*-DKO and *Gak*/*Gadd45b*-DKO mice (Figure 9, E and F).

## Discussion

Previous studies, including ours, have suggested the importance of the clathrin-mediated endocytic pathway in maintaining the integrity of the glomerular filtration barrier (1, 32–35). In order to examine the clinical relevance of endocytosis in human disease, we probed a human database, Nephroseq, and discovered that decreased glomerular expression of clathrin-mediated endocytic pathways correlated with the reduction in GFR. We specifically examined Gak, an important mediator of clathrin coat removal, and used an unbiased screen to identify aberrant calpain activity. The inimical role of elevated calcium in podocytes has been implicated in the development and progression of glomerular kidney diseases (36, 37), and in line with these findings, elevated calcium levels in podocytes from *Gak*-KO mice had elevated calcium and increased calpain-1 and calpain-2 activation driving the injury phenotype. Administration of calpain-1/-2 inhibitors, or targeted deletion of the calpain-1/-2 heterodimer’s common regulatory domain, *Capns1*, reduced both podocyte injury and damage to the glomerular filtration barrier. Endocytosis can be controlled and triggered by calcium channels at the cell membrane and intracellular calcium concentrations (38, 39), suggesting that impairment in endocytosis induced by the loss of *Gak*, similar to the loss of *Dnm*1/2 inhibiting angiotensin II receptor type 1 internalization, results in the dysregulation of cytoplasmic calcium homeostasis (32) and following calpain activation, explaining the observed increase. However, it is unclear in our current study whether loss of podocyte-associated *Gak* directly leads to calpain activation because podocyte injury in itself increases calcium levels (37). Future studies will be needed to further define the exact mechanism. Another possible explanation of increased calpain-1/-2 activities could be the downregulation of the endogenous calpain inhibitor calpastatin (40). Moreover, calpeptin, a calpain inhibitor, markedly attenuates rat proteinuria induced by adriamycin administration in an experimental model for human FSGS (13). In podocytes, calpain has also been associated with endoplasmic reticulum stress-induced proteinuria (16), while urine samples from FSGS patients show a sharp increase in calpain activity compared with healthy controls (15). These findings concomitantly suggest that aberrantly increased calpain activity may drive kidney disease in rodent models and in humans by damaging the podocyte and disrupting glomerular filtration barrier integrity. Our results demonstrate that treatment with CI provided a striking improvement in the progression of kidney disease and in the life span in *Gak*-KO mice. Furthermore, treatment was effective in inhibiting podocyte injury following NTS-induced glomerular injury. In other organ systems, selective loss of *Capns1* in the pulmonary artery smooth muscle cells displays a protective effect in pulmonary hypertension and lung damage (22), suggesting that inhibiting this pathway may provide an avenue for novel therapeutics.

RNA profiling of *Gak*-KO mice glomeruli samples permitted investigation of the site of calpain activation. Genetic ablation of podocyte-associated calpain recapitulated the findings observed in the calpain inhibitor studies. Our microarray data also demonstrated that *Gadd45b* may also play a role downstream of calpain activity. Previous analysis of the baseline podocyte translational profile, using the translating ribosome affinity purification technique on *Actn4^–/–^* mice with FSGS, demonstrates significantly upregulated *Gadd45b* expression (41). Overexpression of zebrafish *Gadd45b* in podocytes has previously been shown to accelerate proteinuria, foot process effacement, and podocyte apoptosis, via the P38 activation pathway (42). Further, the loss of Dicer in mice also causes progressive glomerulosclerosis and kidney failure, with a marked increase in *Gadd45b* expression as identified by microarray analyses of injured podocytes (43). Such findings indicate that increased *Gadd45b* may potentially be involved in podocyte injury. In line with these findings, our results show that loss of *Gadd45b* in the *Gak*-KO mice improved albuminuria and kidney function.

The dysregulated NF-κB signaling pathway may play a critical role because 3 NF-κB binding sites in the *Gadd45b* promoter region can mediate transcriptional activation (27). Among NF-κB subunits, RelA/p65 was observed to be sufficient to activate *Gadd45b* expression (27, 44). Loss of podocyte *Gak* resulted in an increase in phospho-NF-κB p65 bound to the *Gadd45b* promoters, as revealed by ChIP assay. Simultaneous deletion of *Gak* and *Capns1* strongly reduced phospho-NF-κB p65 binding to the *Gadd45b* promoters, suggesting that increased NF-κB p65 activity in the podocyte contributed to the increase in *Gadd45b* expression. Interestingly, constitutional activation of the NF-κB p65 pathway in murine podocytes has been implicated to induce glomerulosclerosis, proteinuria, and progressive kidney failure (45). IκBα is one of the most important substrates of calpain-1/-2; its cleavage and degradation is required for consequent NF-κB translocation to the nucleus (24, 26, 46). In *Gak*-KO mice podocytes, we found increased NF-κB p65 activity and a striking increase in IκBα cleavage; these phenotypes were summarily abrogated by loss of podocyte-associated *Capns1*. Given these findings, we posit that the improvement of glomerulosclerosis and kidney function observed following podocyte-specific deletion of *Capns1* is partially associated with the restoration of IκBα, which in turn inhibits the overactivated NF-κB p65/GADD45B pathway. Proper podocyte adhesion and an intact actin cytoskeleton are critical in maintaining a functional glomerular filtration barrier. Podocyte-specific deficiency in *Gak* appears to reduce podocyte numbers and adhesion, phenotypes that were rescued by the loss of podocyte-specific *Gadd45b* and *Capns1*, respectively. Previously, *Trpc6*-KO podocytes with diminished calpain activity have been observed to have reduced podocyte detachment compared with those podocytes with higher calpain activity (12). Calpain activity has also been implicated to regulate actin cytoskeletal dynamics by targeting many cytoskeleton-associated substrates, including talin (28), paxillin (28), vinculin (47), focal adhesion kinase (28), and integrins (48). Podocyte-specific deficiency in *Gak* also showed dramatic disorganization of the F-actin cytoskeleton.

In summary, we provide evidence that the reduction of human glomerular expression of *Gak* correlates with the decrease in GFR in patients with CKD. We also demonstrated that *Gak* is required in maintaining the integrity of the glomerular filtration barrier using a podocyte-specific loss of this gene. We lastly revealed that podocyte-associated calpain-1/-2 activations orchestrate kidney injury through the NF-κB p65/GADD45B pathway, resulting in podocyte loss. These findings motivate further studies to investigate the different contributions of podocyte-associated calpain 1 versus calpain 2 activity in the pathogenesis of proteinuric kidney disease along with the mechanisms of how the loss of endocytosis leads to increased calcium. Identifying target-specific pathways will be paramount for potential future therapeutic targets in glomerular diseases that display aberrantly elevated calpain activity.

## Methods

### Antibodies, reagents, and plasmids.

Mouse anti-GAK antibody (Santa Cruz Biotechnology, catalog: sc-137066); rabbit anti-GAK antibody (Invitrogen, Thermo Fisher Scientific, catalog: PA5-15321); mouse anti–transducin-like enhancer of split 4 (TLE4) antibody (Santa Cruz Biotechnology, catalog: sc-365406); rabbit anti-IκBα (Santa Cruz Biotechnology, catalog: sc-371); rabbit anti–NF-κB p65 (Cell Signaling Technology, catalog: 8242); rabbit anti-GADD45B antibody (Abcam, catalog: ab105060); rabbit anti–phospho-NF-κB p65 (Ser536) (Cell Signaling Technology, catalog: 3033); rabbit anti-WT1 antibody (Abcam, catalog: ab89901); mouse anti-WT1 antibody (MilliporeSigma, catalog: 05-753); rabbit anti-CAPNS1 antibody (MilliporeSigma, catalog: HPA006872); guinea pig anti-nephrin antibody (Progen, catalog: GP-N2); mouse anti-talin1 antibody (Cancer Research Technology, clone: 97H6); rabbit anti–β-actin antibody (Cell Signaling Technology, catalog: 5174); Alexa Fluor 488–conjugated phalloidin (Invitrogen, Thermo Fisher Scientific, catalog: A12379); Alexa Fluor 594–conjugated phalloidin (Invitrogen, Thermo Fisher Scientific, catalog: A12381); Alexa Fluor 488–conjugated goat anti–mouse IgG antibody (Invitrogen, Thermo Fisher Scientific, catalog: A-11001); Alexa Fluor 594–conjugated goat anti–rabbit IgG antibody (Invitrogen, Thermo Fisher Scientific, catalog: A-11037); Alexa Fluor 594–conjugated goat anti–guinea pig IgG antibody (Invitrogen, Thermo Fisher Scientific, catalog: A-11076); DAPI/antifade solution (MilliporeSigma, catalog: S7113); mouse anti–mouse IgG horseradish peroxidase–conjugated (HRP-conjugated) antibody (Rockland, catalog: 18-8817-31); and rabbit anti–mouse IgG HRP-conjugated antibody (MilliporeSigma, catalog: AP160P) were purchased commercially. Cell culture media were purchased from Invitrogen, Thermo Fisher Scientific. Collagen type I was purchased from BD Biosciences. ChromPure rabbit IgG (catalog: 011-000-003) and complete Freud’s adjuvant (CFA) were purchased from Jackson ImmunoResearch Laboratories and MilliporeSigma, respectively. Fluorogenic calpain-1/-2 substrates (Succinyl-Leu-Tyr-AMC, AMC indicates 7-amino-4-methylcoumarin) were purchased from Calbiochem. Calpain activity assay kit was purchased from Abcam (catalog: ab65308). CI was purchased from Calbiochem (catalog: N-1535). Diphtheria toxin was from MilliporeSigma (catalog: D0564).

### Generation of mice.

*Gak^fl/fl^* mice on C57BL/6 background, in which essential coding exon 1 is flanked by *loxP* sites, were obtained in-house (NIH, Bethesda, Maryland, USA) (49). *Capns1^fl/fl^* mice on C57BL/6 background have essential coding exon 9 to exon 11 flanked by *loxP* sites (Queen’s University, Ontario, Canada) (50). *Gadd45b*^–/–^ mice on a C57BL/6 background were obtained in-house (Temple University, Philadelphia, Pennsylvania, USA) (51, 52). These mice were mated with the *Pod-Cre-Rosa-Dtr^fl^* mice with a DTR knockin on a C57BL/6 background, a gift from Lloyd Cantley (Yale University, New Haven, Connecticut, USA) (18) to generate *Gak^fl/fl^ Pod-Cre-Rosa-Dtr^fl^* (*Gak*-KO) mice, *CAPNS1^fl/fl^* *Pod-Cre-Rosa-Dtr^fl^* (*CAPNS1*-KO) mice, *Gadd45b^–/–^ Pod-Cre-Rosa-Dtr^fl^* (*Gadd45b*-KO) mice, *Gak^fl/fl^ Capns1^fl/fl^ Pod-Cre-Rosa-Dtr^fl^* (*Gak/Capns1*-DKO) mice, and *Gak^fl/fl^ Gadd45b^–/–^ Pod-Cre-Rosa-Dtr^fl^* (*Gak/Gadd45b*-DKO) mice. Cre-negative and *Gadd45b^+/+^* mice were used as controls. All generated mice were backcrossed more than 10 times before experiments. Genotyping by tail prep and PCR were performed at 2 days of age. The genotyping primer sequences were used as follows: *Cre* forward: 5′-ACAGCTCCACCAAGACACAG -3′; *Cre* reverse: 5′-TCCGGTTATTCAACTTGCACC-3′; *Dtr* forward: 5′-CACTGGATCTACGGACCAGC-3′; *Dtr* reverse: 5′-CGATTTTCCACTGGGAGGCT-3′; *Gak* forward: 5′-TGCCCTTTCAGCTCCCACACTCTTCTTCAC-3′; *Gak* reverse: 5′-GCAGCCAGCCTATGAGCCACAGACACTTAT-3′; *Capns1* forward: 5′-GAACTTCCAGGGGCCTTTGAG-3′; *Capns1* reverse1: 5′-GTTTGGTCTCAGGGCCCCAGC-3′; *Capns1* reverse2: 5′-GGTGGGGTGACCCTTCAGTAG-3′; *Gadd45b* forward1: 5′-GCTGTGGAGCCAGGAGCAGCA-3′; *Gadd45b* forward2: 5′-AAGCGCATGCTCCAGACTGCCTT-3′; and *Gadd45b* reverse: 5′-ATATGCAAGCGATCTGTCTTGCTCA-3′.

For the calpain-inhibitory experiment in vivo, CI (20 mg/kg body weight per day, i.p.) was injected into *Gak*-KO mice starting from 2 weeks old with evident albuminuria. The glomerular injury animal model induced by NTS (23) (Lampire Biological Laboratories) was performed as previously described in our laboratory (15). Briefly, 5 days after an i.p. injection of 250 μg of rabbit IgG (Jackson ImmunoResearch Laboratories) in 250 μL of 1:1 emulsion with CFA (MilliporeSigma), NTS (150 μL) was injected by i.v. for 3 days into control C57BL/6 mice at 8 weeks of age, with or without CI administration 2 days after NTS injection. Urine samples were collected at different time points for the determination of calpain-1/-2 activities and urine albumin concentration, respectively. The glomeruli samples were isolated for calpain-1/-2 activities assay, and kidney sections were prepared for the histological analysis.

### Biochemical measurements, including plasma creatinine, urine albumin, and urine creatinine.

Mouse urine samples were collected at various ages. Urine albumin levels were quantitated using an albumin ELISA quantitation kit according to the manufacturer’s protocol (Bethyl Laboratories Inc.), and the absorbance was read at 450 nm (Glomax multi detection system; Promega). Urine albumin and plasma creatinine levels were measured in duplicate for each sample at the time points above using a colorimetric quantification kit (Bioassay Systems) at an absorbance of 490 nm (iMark Microplate Absorbance Reader; Bio-Rad, model 550).

### Cell culture.

Isolation of podocytes from day 7 or day 14 in control mice and *Gak*-KO, *Capns1*-KO, and *Gak*/*Capns1*-DKO mice were performed as described previously in our laboratory (15). Two days after the glomerular cells were seeded on the collagen type I–coated cell culture dishes, medium containing diphtheria toxin (200 ng/mL) was changed every other day until all cells except primary podocytes were dead. The purity of primary podocytes was confirmed by WT1 staining, ensuring that the purity was greater than 98%. Primary podocytes of passage 1 or 2 were used in all experiments.

### Podocyte intracellular calcium concentration measurement.

The measurement of basal intracellular Ca^2+^ ([Ca^2+^]_i_) within the freshly isolated primary podocytes from both control mice and *Gak*-KO mice was performed as previously described with some modifications (53, 54). Briefly, primary culture podocytes were seeded in glass-bottomed, 35 mm dishes (No. 1.5 thickness, MatTek) that were coated with collagen type I and incubated with 2 μM Fluo-4 AM (acetoxymethyl) (excitation at 488 nm, emission at 520 nm) and 0.04% pluronic F127 (Invitrogen, Thermo Fisher Scientific) at 37°C in a 5% CO_2_ incubator for 15 minutes. The cells were washed with Hanks’ balanced salt solution (Gibco, Thermo Fisher Scientific) 3 times and incubated for 10 minutes at 37°C to allow cleavage of intracellular AM esters. The cells were replaced with an imaging buffer, containing 136 mM NaCl, 2.5 mM KCl, 2 mM CaCl_2_, 1.3 mM MgCl_2_ and 10 mM HEPES (pH 7.4). Images were taken in a time series (5 seconds per frame) by an Andor CSU-WDi spinning disc confocal microscope equipped with a Nikon Eclipse Ti-E CFI Plan Apochromat Lambda ×10 objective lens for 8 minutes. After recording the basal signal intensity for 1 minute (F), 10 μM ionomycin (Thermo Fisher Scientific, catalog: 124222) was applied and caused peak fluorescence (F_max_); this was then followed by quenching with 5 mM MnCl_2_ (F_min_) (MilliporeSigma, catalog: 244589). The Fluo-4 AM fluorescence signals within the podocyte were calculated using NIH ImageJ software and the [Ca^2+^]_i_ within each podocyte was calculated using the formula: [Ca^2+^]_i_ = *K_D_* (F – F_min_) /(F_max_ – F), where *K_D_* is the dissociation constant for Fluo-4 AM; F is the intensity at the baseline; and F_min_ and F_max_ are the intensities at the point of maximum Fluo-4 AM fluorescence signal after adding ionomycin and the minimum Fluo-4 AM fluorescence signal after quenching with MnCl_2_, respectively.

### Microarray of glomerular RNA and bioinformatics analysis.

Glomeruli were isolated from the kidney cortical tissue in the control mice and *Gak*-KO mice at 3 weeks of age using Dynabeads (Invitrogen, Thermo Fisher Scientific) perfusion and Percoll density gradient centrifugation (GE Healthcare) as previously described (15). The glomerular RNA was extracted using an RNeasy Plus Mini kit (QIAGEN), and that was sent to the Yale Center for Genome Analysis for microarray analysis on the Affymetrix mouse genome 430 2.0 platform.

The raw microarray gene expression signals were analyzed by the R Bioconductor package as previously described (55). Briefly, probes were mapped to gene symbols for each data set using the annotation file downloaded from the Affymetrix website; probes without annotations were discarded. The *Z*-ratio of each gene was calculated by comparing its expression in different samples. A *Z*-ratio value greater than 1.96 was considered indicative of DEGs and selected for further analysis; these were shown in a 2-dimensional hierarchical heatmap. The genes in the heatmap colored red and blue indicated the upregulated and downregulated genes, respectively. IPA (QIAGEN) was used to assess biological relations among the DEGs from control and *Gak*-KO mice (*n* = 6 mice) glomeruli microarray data sets. The pathway was generated by IPA on known signaling and metabolism pathways available. The Gene Expression Omnibus accession number is GSE158384.

### Quantitative PCR analysis.

Total RNA was extracted from the isolated glomeruli using an RNase kit (QIAGEN). The RNA concentration was measured by spectrophotometry NanoDrop ND2000c (NanoDrop Technologies, Thermo Fisher Scientific), and any sample with OD260/OD280 greater than or equal to 2.0 was used for the experiments. From each sample, 1 μg of RNA was used for reverse transcription to cDNA using a high-capacity cDNA synthesis kit following the manufacturer’s instructions (Applied Biosystems, Thermo Fisher Scientific). All quantitative PCR amplifications were performed using Power SYBR Green Master Mix with a 7300 AB real-time PCR machine (Applied Biosystems, Thermo Fisher Scientific). Mouse *Gapdh* gene expression was used as the internal control for each sample’s relative gene expression. The real-time PCR condition is as follows: 95°C for 3 minutes, then 95°C for 10 seconds and 58°C for 30 seconds, 40 cycles. The primer sequences are as follows: *Gapdh* forward 5′-TCACCACCATGGAGAAGGC-3′; *Gapdh* reverse 5′-GCTAAGCAGTTGGTGGTGCA-3′; *Serpinb6* forward 5′-GCTATTTGAGGCCAGAGCACAG-3′; *Serpinb6* reverse 5′-GGAGCTGTCTTCACCCAGTGTT-3′; *Anxa1* forward 5′-GATCAAGGCCGCGTACTTACA-3′; *Anxa1* reverse 5′-GCTGGAGTTTTTAGCATAGCCA-3′; *Gadd45b* forward 5′-TTGACATCGTCCGGGTATCAG-3′; *Gadd45b* reverse 5′-GTCTCGGGCTTCGGTTGTG-3′; *Abra* forward 5′-GCCAGGATCAAACGCCCCT-3′; *Abra* reverse 5′-GCTGCCACCTCCCTTTCAAGT-3′; *Itga2* forward 5′-TCCGGGCCTTCAAGTGAACA-3′; *Itga2* reverse 5′-CGGTTCTCAGGGAAGCCACT-3′; Ankrd1 forward 5′-TTGATGACCTTCGGTGCGGA-3′; Ankrd1 reverse 5′-GCTGGAGTTTTTAGCATAGCCA-3′; *Bcl3* forward 5′-CCGGAGGCCCTTTACTACCA-3′; *Bcl3* reverse 5′-GGAGTAGGGGTGAGTAGGCAG-3′; *Fn1* forward 5′-GGAGGCACTGCAGAACCAGA-3′; *Fn1* reverse 5′-TGGTTCAGGCCTTCGCTGA-3′; *Kap* forward 5′-AGTCTCCTCCGGCTTTCTGGA-3′; *Kap* reverse 5′-AGTAGGGGAGACTGGATTCCCA-3′; *Egf* forward 5′-TTGACAAGTGGCAGGAGGTC-3′; *Egf* reverse 5′-CACCCAAGAGTACAGCCGTGA-3′; *Gabrb3* forward 5′-CAATCTGGCTTTCCTGGACCCT-3′; *Gabrb3* reverse 5′-CAACAGCTTGTCGACCGTCTC-3′; *Slc22a22* forward 5′-AGACTTGCACCATTGGGTCAG-3′; and *Slc22a22* reverse 5′-CCATCATGCGGCATAGGACA-3′.

### Western blot.

Primary podocytes or freshly isolated glomeruli were lysed in lysis buffer containing 50 mM Tris-HCl (pH: 7.6), 500 mM NaCl, 0.1% SDS, 0.5% deoxycholate, 1% Triton X-100, 0.5 mM MgCl_2_, a protease inhibitor cocktail (Roche Diagnostics), and a phosphatase inhibitor cocktail (MilliporeSigma). Protein concentrations were quantified using a Bio-Rad protein assay. The equal 20 to 40 μg amounts of protein were denatured for 10 minutes at 95°C, and samples were loaded to each lane and separated by 4%–20% gradient SDS-PAGE gels and transferred to a PVDF membrane (MilliporeSigma). The membrane was blocked with 5% nonfat milk (American Bio) or 3% BSA (MilliporeSigma) in Tris-buffered saline and 0.05% Tween-20 (TBST) and incubated with appropriate primary antibodies at 4°C on a shaker overnight. After 3 washes with 1× TBST, the appropriate HRP-conjugated secondary antibodies were added, and signals were detected using enhanced chemiluminescence reagents (Bio-Rad). The blots were then exposed using Odyssey (LI-COR Biosciences). For quantification, densitometry was performed using the NIH ImageJ software.

### ChIP assay.

The ChIP assay was performed using anti–phospho-NF-κB p65 antibody with a SimpleChIP kit as per the manufacturer’s protocol (Cell Signaling Technology). Briefly, freshly isolated primary podocytes were cross-linked with 1% formaldehyde for 10 minutes. The chromatin of cells was extracted by sonication to generate chromatin fragments between 150 and 900 bp (Misonix sonicator). Immunoprecipitation of phosphorylated NF-κB p65–cross-linked chromatin was performed using magnetic Dynabeads coated in rabbit anti–phospho-NF-κB p65 antibody. Normal sheep anti–rabbit IgG was used as the control for nonspecific IgG binding. Coprecipitated DNA and input DNA were used for the analysis of the *Gadd45b* promoter region by reverse transcriptase PCR. The analysis was conducted by real-time PCR using SYBR Green qPCR Supermix. The final result was calculated as a ratio of each sample’s coprecipitated DNA and input DNA PCR signals. PCR primers were designed from previously published sequences for the promoter region of mouse *Gadd45b* gene, as follows, forward: 5′-ATGCACATCCCTTCTTTCAGAGC-3′; reverse: 5′-CGCGCGGGGTTTCCAG-3′ (27).

### Adhesion assay.

The adhesion assay with crystal violet staining was performed as previously described (15). Briefly, freshly isolated primary podocytes were trypsinized, and cells were counted by adding trypan blue using an automated cell counter (Logos Biosystems, Inc.; model: LUNA-FL). Equal amounts of the alive cells in each well were seeded on a 96-well plate coated with collagen type I (10 mg/mL) and allowed to attach for 2 hours at 37°C in a 5% CO_2_ humidified incubator. Nonadherent cells were removed by gentle washing with 1× PBS. It was followed by fixation in 95% ethanol for 20 minutes. Fixed cells were stained with 0.1% crystal violet (MilliporeSigma) for 15 minutes at room temperature, washed gently in distilled water 3 times in order to remove the unstained crystal violet, and lysed in 0.2% Triton X-100 for shaking until a uniform color was obtained. The absorbance of dissolved crystal violet was measured using a microplate reader at 595 nm (Bio-Rad).

### Calpain-1/-2 activities assay.

For freshly isolated primary podocytes or freshly isolated glomeruli, calpain-1/-2 activities were determined using a calpain activity assay kit (Abcam, catalog: ab65308) according to the manufacturer’s protocol with some modifications. Briefly, clarified podocyte lysate or homogenized glomeruli lysate (mixed with lysis buffer provided with the kit) were incubated with 80 μM fluorogenic calpain-1/-2 substrate (succinyl-Leu-Tyr-AMC, where AMC indicates 7-amino-4-methylcoumarin) (MilliporeSigma) and reaction buffer for 1 hour at 37°C in the dark. Upon cleavage of the substrate by calpain, the fluorogenic portion of AMC was detected at 365 nm excitation and 460 nm emission (Glomax Multi Detection System; Promega). Calpain-1/-2 activities in each sample were expressed as relative fluorescence units (RFUs) per milligram of protein per minute in the absence of CI, a specific inhibitor to the calpain-1/-2 activities, subtracted from the same sample in the presence of CI. For measuring calpain activity in urine samples, calpain-1/-2 activities in each sample were expressed as RFU/μg of urine creatinine concentration per minute in the absence of CI, subtracted from the same sample in the presence of CI.

*NF-κB p65activity assay*. The nuclei protein of freshly isolated podocytes was extracted using NE-PER nuclear and cytoplasmic extraction reagents (Thermo Fisher Scientific, catalog: 78835) according to the manufacturer’s protocol. NF-κB p65 activity of the nuclei protein sample was measured using the NF-κB p65 Transcription Factor Assay Kit (Abcam, catalog: ab133112) per the manufacturer’s protocol. The NF-κB p65 activity in each sample was expressed as absorbance units at 450 nm (OD)/μg of cell nuclei extract.

### Kidney histology and quantification.

Mice were anesthetized via i.p. injection of ketamine and xylazine, followed by perfusion fixation with 4% paraformaldehyde (with or without 2% glutaraldehyde) injected through the left ventricle. The 4 μm kidney sections were then analyzed for histopathology changes with a light microscope and ultrastructural changes with the TEM, respectively. Sections were sent to the Yale Pathology Core Tissue Service for H&E, PAS, and Masson’s trichrome staining. To assess glomerulosclerosis and renal tubulointerstitial lesions, kidney sections with Masson’s trichrome staining were assessed as previously described (15, 56). Briefly, the severity of glomerulosclerosis in each glomerulus was scored in a double-blinded manner as follows: 0 = no sclerosis; 1 = sclerosis of <10% of the glomeruli; 2 = sclerosis of 10%–25% of the glomeruli; 3 = sclerosis of 25%–50% of the glomeruli; 4 = sclerosis of >50% of the glomeruli. A total of 40 glomeruli of each mouse kidney tissue were examined as the PAS-stained section. To evaluate the renal tubulointerstitial lesions — defined as tubular dilation, tubular atrophy, cast formation, and interstitial fibrosis — 20 fields of each mouse kidney tissue were examined as Masson’s trichrome–stained sections. Semiquantitative analysis was scored as follows: 0 = no lesion; 1 = lesion of <10% of the areas; 2 = lesion of 10%–25% of the areas; 3 = lesion of 25%–50% of the areas; 4 = lesion of >50% of the areas. The number of foot processes in each capillary of the glomerulus and the thickness of GBM were measured using the NIH ImageJ software as previously described (15).

### Immunofluorescence staining.

Freshly isolated primary podocytes seeded on collagen type I–coated coverslips were washed with 1× PBS and fixed with 4% paraformaldehyde for 20 minutes at room temperature and then permeabilized with 0.1% Triton X-100 for 20 minutes at room temperature (RT). Human kidney biopsy paraffin-embedded sections were first deparaffinized with xylene and then hydrated with a decreasing gradient concentration of ethanol. For human kidney biopsy paraffin-embedded sections and mouse cryosections, antigen retrieval was performed with sodium citrate buffer (10 mM sodium citrate, 0.05% Tween-20, pH 6.0) at 95°C for 10 minutes. The 4 μm kidney sections or primary podocytes on the coverslips were blocked with 3% BSA in 1× PBS for 1 hour at RT, then incubated with the primary antibodies at 4°C overnight. The following day, slides were incubated with the appropriate Alexa Fluor 488– and/or Alexa Fluor 594–conjugated secondary antibodies for 1 hour in the dark at RT, then mounted with Slowfade (Invitrogen, Thermo Fisher Scientific). Images were taken by an Andor CSU-WDi spinning disc confocal microscope equipped with a Nikon Eclipse Ti-E CFI Plan Apochromat Lambda ×60 oil immersion objective for immunofluorescence analysis. Images were analyzed using NIH ImageJ software or Adobe Photoshop CS6.

For quantification of GAK expression in the human kidney biopsy tissues, GAK staining signal intensity in each selected glomerulus region was measured for the integrated intensity (I_GAK_) and area (A), and background intensity was measured by averaging the 3 different areas closest to the defined glomerulus in the same slide without specific staining (I_BK_). The corrected intensity of GAK expression of each glomerulus was determined by subtracting I_BK_ × A from I_GAK._

For quantification of podocyte numbers, WT1-positive nuclei per glomerular tuft on mouse kidney tissue, or TLE4-positive nuclei per glomerular tuft on human kidney biopsy tissue, were counted as previously reported. TLE4 is a transcriptional corepressor factor, which was used as an alternative podocyte nuclear marker since the WT1 may be less robustly expressed in formalin-fixed sections of the human kidney (57).

For quantification of changes in the actin cytoarchitecture of primary podocytes, podocytes stained with phalloidin were grouped into 4 classes as follows and were scored as previously described (15): type A — more than 90% of cell area filled with thick cables; type B — at least 2 thick cables running under nucleus and rest of cell area filled with fine cables; type C — no thick cables but some cables present; type D — no cables visible in the central area of the cell.

### Statistics.

All data were displayed as mean ± SEM. The number of replicates for each experiment is described above or in the figure legends. Statistical analysis was performed using a 2-tailed *t* test for differences between 2 groups’ comparisons, or 1-way ANOVA followed by Dunnett’s multiple comparisons tests, in order to better account for differences among multiple-group comparisons; survival curves were created and compared by a log-rank test (GraphPad Prism 8 software). A *P* value less than 0.05 was considered statistically significant.

### Study approval.

All animal experiments were approved by Yale University’s Yale Animal Resource Center protocol 2019-11196. All work was carried out in accordance with the principles and procedures outlined in the NIH guidelines for the care and use of experimental animals. All human renal biopsy specimen slides of FSGS and healthy patients were obtained from the Department of Pathology at Yale and Johns Hopkins, and the protocol was approved by the Yale-New Haven Hospital’s Institutional Review Board and Johns Hopkins Institutional Review Board, respectively.

## Author contributions

XT, KI, YW, JS, CEP, TZ, MY, and MG performed the experiments and statistical analysis. YZ and ZZ performed the microarray analysis. DAL, LG, PG, DGM, CRP, and SI designed the experiments, and XT, KE, WL, and SI wrote the manuscript.

## Supplementary Material

supplemental data

supplemental Table 1

## Version 1 11/19/2020

Electronic publication
