# Supplementary material for: Inhibiting calpain 1 and 2 in cyclin G associated kinase–knockout mice mitigates podocyte injury
Source: JCI Insight. 2020 Nov 19;5(22):e142740. doi: 10.1172/jci.insight.142740 (PMC7710277; doi:10.1172/jci.insight.142740)
Supplement: supplemental data [file jciinsight-5-142740-s160.pdf]

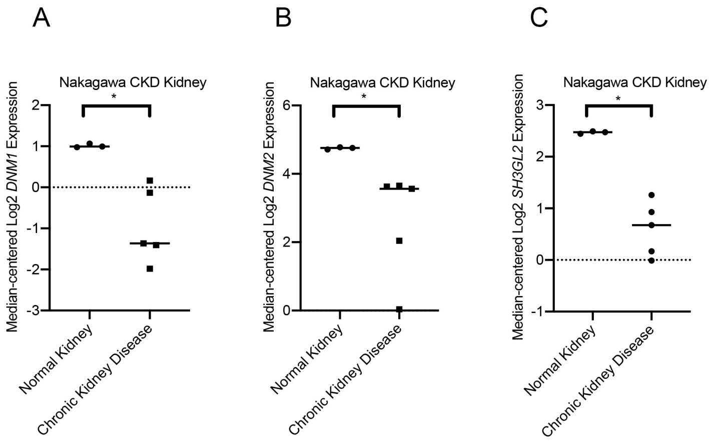

Supplemental Figure 1. *DNM1*, *DNM2*, and *SH3GL2*, expression in CKD from Nephroseq datasets. (A) *DNM1* expression in the glomeruli from normal and CKD patients. Fold change=-3.877. **(B)** *DNM2* expression in the glomeruli from normal and CKD patients. Fold change=-4.480. **(C)** *SH3GL2* expression in the glomeruli from normal and CKD patients. Fold change=-3.648. *p<0.01

Supplemental Figure 2

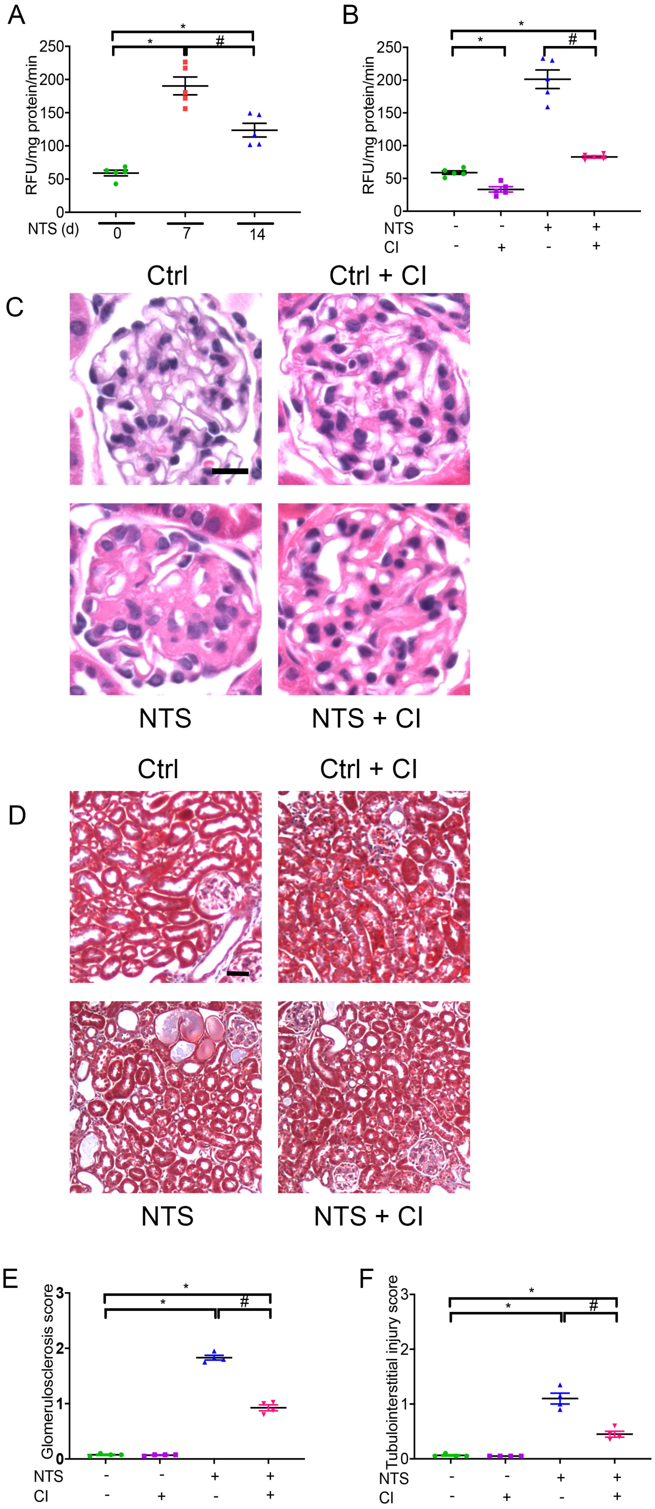

Supplemental Figure 2. Calpain inhibitor III (CI) improves the kidney injury and albuminuria in mice treated with NTS administration, a podocyte injury animal model. (A) The calpain-1/-2 activities from freshly isolated glomeruli in NTS treated mice at 7 and 14 days. n=5. **(B)** The calpain-1/-2 activities from the freshly isolated glomeruli in NTS treated mice +/- CI at 7 days. n=5. **(C)** Representative light microscope images (PAS) of glomeruli from control, control treated with CI, NTS, and NTS treated with CI (20 mg/kg body weight daily by I.P). 14 days after treated with NTS. Scale bar=25 µm. n=4. **(D)** Representative trichrome staining in control, control treated with CI, NTS, and NTS treated with CI. Scale bar=100 µm. **(E)** Quantification of glomerulosclerosis in (C)**,** n=4 * P<0.05 vs control mice, # P<0.05 vs NTS mice. **(F)** Quantification of tubulointerstitial injury in (D), n=4 * P<0.05 vs control mice, # P<0.05 vs NTS mice. (A, B, E, and F) Statistically analyzed via a one-way ANOVA with Dunnett's correction.

Supplemental Figure 3

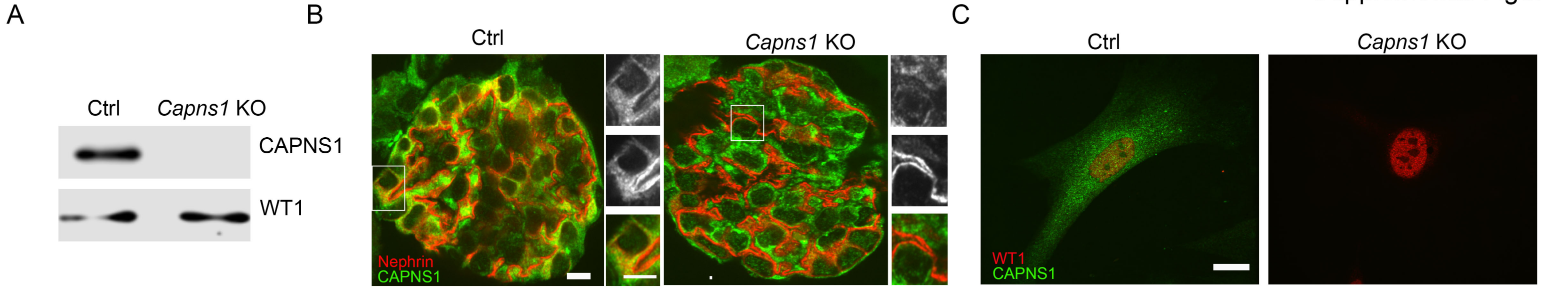

Supplemental Figure 3. Generation of *Capns1* KO mice. (A) Representative immunoblots of CAPNS1 and WT1 in primary podocytes from the control, *Capns1* KO mouse at P7. **(B-C)** Representative immunofluorescence of CAPNS1 and nephrin in kidney sections (B) and CAPNS1 and WT1 in primary podocytes (C) from the control and *Capns1* KO mouse at P7. Scale bar= 10µm.

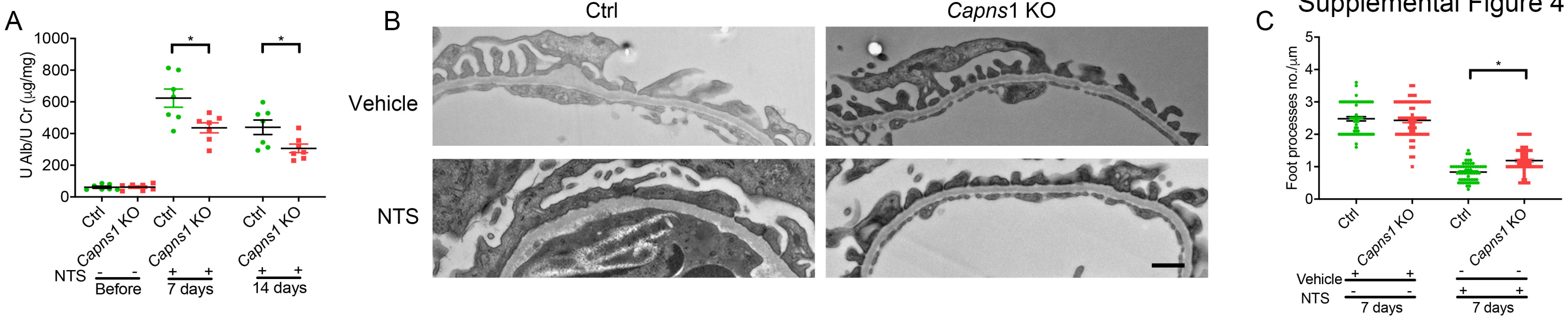

Supplemental Figure 4. ***Capns1*** **KO mice have reduced albuminuria and foot process effacement following NTS administration. (A)** Urine albumin/creatinine ratio in control and *Capns1* KO treated with or without NTS at 7 and 14 days n=7 *P<0.05 vs control mice treated with vehicle. **(B)** Representative TEM image of control and *Capns1* KO mice podocytes with or without NTS. Scale bar= 100μm. **(C)** Quantification of B. n=4 mice per group, 15 glomerular capillary tufts from each mouse were evaluated. * P<0.05 vs control mice treated with vehicle, **(A)** Statistically analyzed by a two tailed *t* test at different time points**. (C)** Statistically analyzed via a one-way ANOVA with Dunnett's correction.

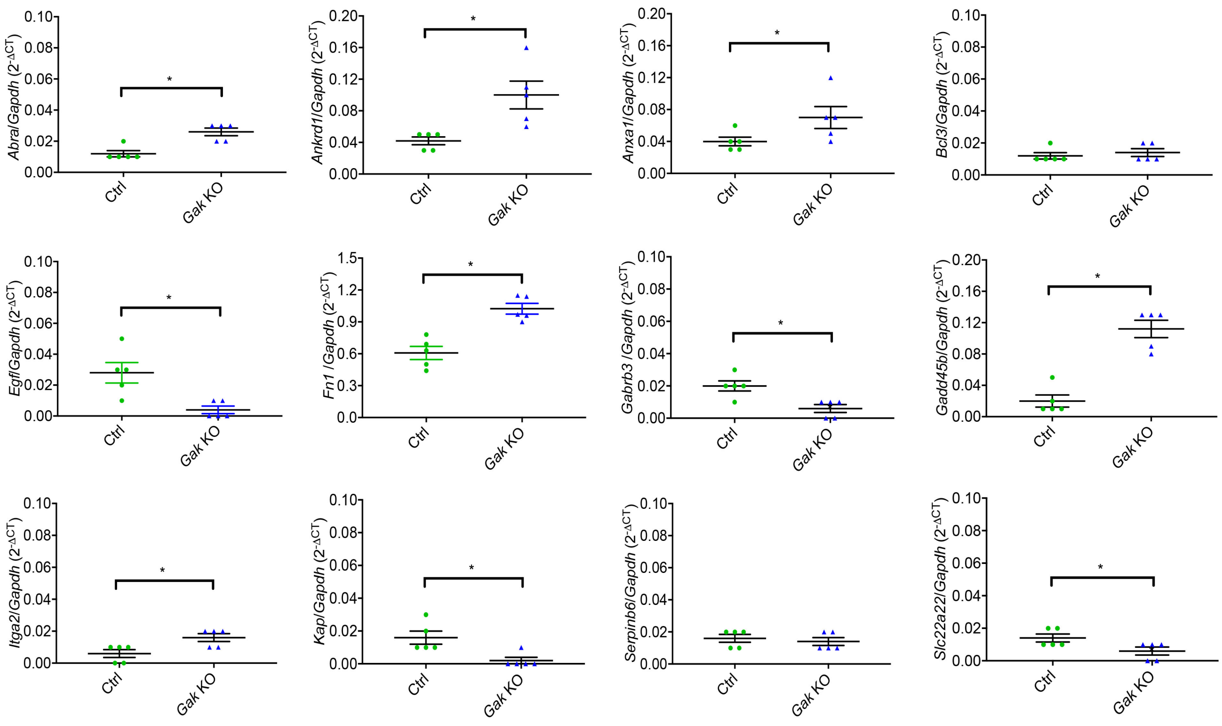

Supplemental Figure 5 Transcriptional levels of differentially expressed candidate genes from glomeruli of control and Gak KO mice. Reverse transcriptase PCR of the candidate genes in control, and Gak KO mice glomeruli. *P < 0.05 vs. control mice *n=5 Statistically analyzed by a two-tailed t test.
