# Supplementary material for: Inhibiting calpain 1 and 2 in cyclin G associated kinase–knockout mice mitigates podocyte injury
Source: JCI Insight. 2020 Nov 19;5(22):e142740. doi: 10.1172/jci.insight.142740 (PMC7710277; doi:10.1172/jci.insight.142740)
Supplement: supplemental Table 1 [file jciinsight-5-142740-s161.pdf]

Supplemental table 1 Gene list from microarray data analysis (*Gak* KO vs control at 3 weeks of age)

Gene_assignment	Protein Name	Gene Symbol	Z ratio (1st batch)	Z ratio (2nd batch)
Z ratioNM_011415	snail family zinc finger 2	Snai2	2.49	2.12
NM_177741	protein phosphatase 1, regulatory (inhibitor) subunit 3B	Ppp1r3b	2.50	2.09
ENSMUST00000103997	predicted gene, 25091	Gm25091	2.46	2.04
ENSMUST00000131387	predicted gene 15963	Gm15963	2.45	2.19
NM_012037	vesicle amine transport protein 1 homolog (T californica)	Vat1	2.27	2.11
AK037717	RIKEN cDNA A130040M12 gene	A130040M12Rik	2.27	2.09
NM_010043	desmin	Des	2.36	2.01
NR_028549	small nucleolar RNA, C	Snord37	2.40	2.40
ENSMUST00000033386	MAS-related GPR, member F	Mrgprf	2.38	2.40
NM_001205313	signal transducer and activator of transcription 1	Stat1	2.45	2.37
NM_009773	budding uninhibited by benzimidazoles 1 homolog, beta (S. cerevisiae)	Bub1b	2.28	2.22
NM_010745	lymphocyte antigen 86	Ly86	2.22	2.27
ENSMUST00000156081	predicted gene 12840	Gm12840	2.27	2.34
NM_145584	spondin 1, (f-spondin) extracellular matrix protein	Spon1	2.07	2.18
NR_045708	predicted gene 11944	Gm11944	1.98	2.00
ENSMUST00000104327	predicted gene, 25878	Gm25878	2.14	2.40
NM_134156	actinin, alpha 1	Actn1	2.09	2.36
NM_181444	G protein-coupled receptor, family C, group 5, member A	Gprc5a	2.06	2.41
NM_023061	melanoma cell adhesion molecule	Mcam	2.03	2.39
NM_175265	bora, aurora kinase A activator	Bora	2.04	2.53
NM_178405	ATPase, Na+	Atp1a2	2.04	2.46
NM_001113179	budding uninhibited by benzimidazoles 1 homolog (S. cerevisiae)	Bub1	2.22	2.22
ENSMUST00000175366	microRNA 1839	Mir1839	2.12	2.78
NR_051981	histocompatibility 2, Q region locus 5	H2-Q5	2.07	2.65
NM_133762	non-SMC condensin II complex, subunit G2	Ncapg2	2.12	3.04
NM_001166727	vomeronasal 1 receptor 175	Vmn1r175	2.11	3.03
NM_181416	Rho GTPase activating protein 11A	Arhgap11a	2.12	2.98

NR_035418	microRNA 669h	Mir669h	2.04	3.00 .
NM_009162	secretogranin V	Scg5	2.01	3.00
ENSMUST00000104091	predicted gene, 22777	Gm22777	2.00	3.08
NM_001013833	protein kinase, cGMP-dependent, type I	Prkg1	1.96	2.91
NM_133903	spondin 2, extracellular matrix protein	Spon2	2.13	3.19
XM_006544010	RIKEN cDNA A630081J09 gene	A630081J09Rik	2.12	3.14
NM_031185	A kinase (PRKA) anchor protein (gravin) 12	Akap12	2.19	3.13
NM_026085	phenazine biosynthesis-like protein domain containing 2	Pbld2	2.39	3.06
NM_001252057	lymphocyte antigen 6 complex, locus C1	Ly6c1	2.34	3.07
NM_008690	nuclear factor of kappa light polypeptide gene enhancer in B cells inhibitor, epsilon	Nfkbie	2.28	3.05
ENSMUST00000103981	predicted gene, 25396	Gm25396	2.23	2.96
NM_007781	colony stimulating factor 2 receptor, beta 2, low-affinity (granulocyte-macrophage)	Csf2rb2	2.35	2.85
NM_021493	Rho GTPase activating protein 23	Arhgap23	2.90	2.51
NM_009255	serine (or cysteine) peptidase inhibitor, clade E, member 2	Serpine2	2.81	2.49
NM_008334	interferon alpha 7	Ifna7	2.79	2.40
NM_205823	toll-like receptor 12	Tlr1	2.66	2.34
NM_001008497	purinergic receptor P2Y, G-protein coupled, 14	P2ry14	2.73	2.44
NM_001101475	RIKEN cDNA F830016B08 gene	F830016B08Rik	2.70	2.47
NM_008415	jerky	Jrk	2.60	2.40
ENSMUST00000146935	predicted gene 20695	Gm20695	2.95	2.68
NM_010821	macrophage expressed gene 1	Mpeg1	2.76	2.70
XM_144599	predicted gene 4963	Gm4963	2.62	2.09
NM_001290397	myeloblastosis oncogene-like 1	Mybl1	2.57	2.06
ENSMUST00000082490	predicted gene, 22749	Gm22749	2.74	2.11
ENSMUST00000181522	predicted gene, 26615	Gm26615	2.94	2.06
NM_009776	serine (or cysteine) peptidase inhibitor, clade G, member 1	Serping1	3.28	2.00
ENSMUST00000122695	predicted gene, 24379	Gm24379	3.22	1.97
XM_006516991	predicted gene 8672	Gm8672	3.19	2.16
NM_001007572	transient receptor potential cation channel, subfamily V, member 5	Trpv5	3.41	2.15
ENSMUST00000082984	predicted gene, 22571	Gm22571	3.37	2.01

ENSMUST00000095920	predicted gene 11360	Gm11360	3.01	2.41
BC091570	kinesin family member C5B	Kifc5b	2.96	2.37
ENSMUST00000158081	predicted gene, 25552	Gm25552	3.04	2.28
NM_013454	ATP-binding cassette, sub-family A (ABC1), member 1	Abca1	3.21	2.48
NM_001001892	histocompatibility 2, K1, K region	H2-K1	3.74	2.94
NM_001290308	collagen, type XII, alpha 1	Col12a1	3.65	2.89
NM_008748	dual specificity phosphatase 8	Dusp8	3.73	2.82
NM_010116	kallikrein 1-related peptidase b9	Klk1b9	3.65	2.73
ENSMUST00000128411	T cell specific GTPase 2	Tgtp2	3.19	3.12
NM_008489	lipopolysaccharide binding protein	Lbp	3.14	2.08
NM_011019	oncostatin M receptor	Osmr	3.78	2.40
ENSMUST00000083225	predicted gene, 25482	Gm25482	3.50	2.27
NR_028282	small nucleolar RNA, C	Snord83b	3.88	2.18
NM_001271676	interferon gamma inducible protein 47	Ifi47	3.75	2.09
NM_001114088	PDZ and LIM domain 7	Pdlim7	2.21	3.66
NM_001243132	tetraspanin 2	Tspan2	2.15	3.61
NM_030704	heat shock protein 8	Hspb8	2.32	3.51
ENSMUST00000104090	predicted gene, 22778	Gm22778	2.20	3.40
ENSMUST00000083274	predicted gene, 24616	Gm24616	2.13	3.37
NM_010729	lysyl oxidase-like 1	Loxl1	2.11	3.50
NR_035474	microRNA 669m-1	Mir669m-1	2.08	3.66
ENSMUST00000122780	predicted gene, 24139	Gm24139	2.00	3.67
NM_008655	growth arrest and DNA-damage-inducible 45 beta	Gadd45b	2.42	3.70
NM_011653	tubulin, alpha 1A	Tuba1a	2.34	3.68
NM_011454	serine (or cysteine) peptidase inhibitor, clade B, member 6b	Serpinb6	2.27	3.87
NM_025427	regulator of cell cycle	Rgcc	2.19	4.16
ENSMUST00000178586	predicted gene, 22887	Gm22887	2.99	3.22
ENSMUST00000076710	predicted pseudogene 7665	Gm7665	2.94	3.26
NM_144899	ADAMTS-like 4	Adamtsl4	3.01	3.31
NR_027872	RIKEN cDNA 4930515G01 gene	4930515G01Rik	2.81	3.21
NM_010217	connective tissue growth factor	Ctgf	2.93	3.64
NM_013912	apelin	Apln	2.83	3.62
XM_006505219	predicted gene 4477	Gm4477	2.65	3.33
ENSMUST00000158685	predicted gene, 23181	Gm23181	2.63	3.31
NM_001029856	ATPase family, AAA domain containing 5	Atad5	2.57	3.31
NR_039562	microRNA 5103	Mir5103	2.62	3.24
ENSMUST00000025595	family with sequence similarity 111, member A	Fam111a	2.46	3.29
ENSMUST00000082648	predicted gene, 25135	Gm25135	3.55	3.72
ENSMUST00000104444	predicted gene, 23266	Gm23266	3.45	3.65
NM_008871	serine (or cysteine) peptidase inhibitor, clade E, member 1	Serpine1	3.55	3.38
NM_033601	B cell leukemia	Bcl3	4.11	3.85
ENSMUST00000122548	predicted gene, 22107	Gm22107	3.93	3.64
ENSMUST00000082683	nuclear encoded rRNA 5S 176	n-R5s176	3.22	4.57
ENSMUST00000158467	predicted gene, 25654	Gm25654	3.15	4.48
NM_010516	cysteine rich protein 61	Cyr61	3.48	4.37
NM_173749	peptidase domain containing associated with muscle regeneration 1	Pamr1	3.19	3.99
NM_178695	proline rich Gla (G-carboxyglutamic acid) 4 (transmembrane)	Prrg4	4.68	2.23
NM_001289492	guanylate binding protein 3	Gbp3	4.77	2.13
ENSMUST00000180104	predicted gene, 24508	Gm24508	4.53	2.21
XR_397994	cDNA sequence BC023105	BC023105	4.71	1.97
NM_001135115	predicted gene 12250	Gm12250	4.15	2.34
NM_001276409	fibronectin 1	Fn1	4.17	2.19

NM_019440	immunity-related GTPase family M member 2	Irgm2	4.58	2.48
NM_008396	integrin alpha 2	Itga2	4.39	2.97
NM_001161730	transporter 1, ATP-binding cassette, sub-family B (MDR)	Tap1	4.45	2.87
ENSMUST00000104797	predicted gene, 23134	Gm23134	4.46	3.30
NM_001161746	tumor necrosis factor receptor superfamily, member 12a	Tnfrsf12a	5.13	3.24
NM_001042605	CD74 antigen (invariant polypeptide of major histocompatibility complex, class II antigen-associated)	Cd74	5.65	2.60
NM_008491	lipocalin 2	Lcn2	5.63	2.48
NM_010104	endothelin 1	Edn1	5.36	2.66
ENSMUST00000043170	predicted gene 9765	Gm9765	5.40	2.02
NM_001146275	interferon inducible GTPase 1	Iigp1	6.44	3.20
NM_172796	schlafen 9	Slfn9	6.13	2.91
XM_003945749	interferon-inducible GTPase 1-like	LOC630751	6.95	3.90
NM_010260	guanylate binding protein 2	Gbp2	7.98	2.95
NM_013468	ankyrin repeat domain 1 (cardiac muscle)	Ankrd1	5.88	6.33
NM_021274	chemokine (C-X-C motif) ligand 10	Cxcl10	6.26	5.52
ENSMUST00000083097	predicted gene, 22364	Gm22364	4.71	5.81
NM_016928	toll-like receptor 5	Tlr5	2.67	5.56
NM_153098	CD109 antigen	Cd109	2.72	5.10
NM_001291894	leukocyte immunoglobulin-like receptor, subfamily B, member 4	Lilrb4	2.26	5.32
NM_001143689	histocompatibility 2, Q region locus 4	H2-Q4	2.54	4.63
NM_010730	annexin A1	Anxa1	2.29	6.13
NM_033325	lysyl oxidase-like 2	Loxl2	3.43	5.14
NM_009367	transforming growth factor, beta 2	Tgfb2	3.15	5.19
ENSMUST00000174960	predicted gene, 24515	Gm24515	3.45	5.54
NR_035452	microRNA 1931	Mir1931	3.84	5.10
NM_009627	adrenomedullin	Adm	2.68	7.64
NM_020025	UDP-Gal:betaGlcNAc beta 1,3-galactosyltransferase, polypeptide 2	B3galt2	2.60	7.09
NM_023485	syncoilin	Sync	2.18	7.93
NM_175456	actin-binding Rho activating protein	Abra	3.12	8.33
NM_009778	complement component 3	C3	13.06	2.14
NR_039546	microRNA 101c	Mir101c	-7.15	-3.09
NM_010594	kidney androgen regulated protein	Kap	-6.21	-4.69
NM_138951	tetratricopeptide repeat domain 36	Ttc36	-2.15	-2.99
NM_001037842	camello-like 3	Cml3	-2.74	-2.85
ENSMUST00000180342	predicted gene, 23862	Gm23862	-2.57	-3.17
ENSMUST00000104034	predicted gene, 22270	Gm22270	-2.84	-3.41
NM_001101640	transmembrane protein 207	Tmem207	-2.87	-3.60
NM_172378	solute carrier family 22 (organic cation transporter), member 22	Slc22a22	-2.36	-3.89
NM_001285881	RIKEN cDNA D630023F18 gene	D630023F18Rik	-2.53	-3.88
NM_019395	fructose bisphosphatase 1	Fbp1	-2.02	-3.59
NM_181588	carboxymethylenebutenolidase-like (Pseudomonas)	Cmbl	-2.29	-3.61
NM_001160097	claudin 10	Cldn10	-2.11	-3.91
ENSMUST00000082798	predicted gene, 23447	Gm23447	-2.04	-3.74
NM_019545	hydroxyacid oxidase 2	Hao2	-2.19	-3.74
NM_007860	deiodinase, iodothyronine, type I	Dio1	-3.04	-2.48
NM_001038701	gamma-aminobutyric acid (GABA) A receptor, subunit beta 3	Gabrb3	-3.28	-2.43
NM_207216	UDP glycosyltransferases 3 family, polypeptide A1	Ugt3a1	-3.85	-2.01
ENSMUST00000157627	small nucleolar RNA, C	Snord13	-3.87	-2.48
NM_001099917	small integral membrane protein 24	Smim24	-1.97	-2.62
NM_025961	glycine amidinotransferase (L-arginine:glycine amidinotransferase)	Gatm	-2.09	-2.55
NM_008287	heat-responsive protein 12	Hrsp12	-1.98	-2.31
NM_028747	thioesterase superfamily member 7	Them7	-2.15	-2.37
NM_133254	solute carrier family 5 (sodium	Slc5a2	-2.19	-2.39
NM_001278605	uromodulin	Umod	-2.44	-2.54

ENSMUST00000174143	predicted gene 11128	Gm11128	-2.56	-2.31
ENSMUST00000143083	predicted gene 10721	Gm10721	-2.65	-2.28
NM_001079690	solute carrier family 12, member 1	Slc12a1	-2.80	-2.30
ENSMUST00000157129	predicted gene, 23470	Gm23470	-2.69	-2.47
ENSMUST00000082907	predicted gene, 26205	Gm26205	-2.48	-2.04
NM_010113	epidermal growth factor	Egf	-3.66	-2.14
ENSMUST00000156693	predicted gene 13773	Gm13773	-2.12	-1.99
NM_025983	ATP synthase, H+ transporting, mitochondrial F1 complex, epsilon subunit	Atp5e	-2.10	-2.23
ENSMUST00000082721	predicted gene, 25788	Gm25788	-2.20	-2.13
NM_001293559	cytochrome c oxidase subunit IV isoform 1	Cox4i1	-2.24	-2.24
